# Using the WHO-INTEGRATE evidence-to-decision framework to develop recommendations for induction of labour

**DOI:** 10.1186/s12961-022-00901-7

**Published:** 2022-11-07

**Authors:** Melissa Murano, Doris Chou, Maria Laura Costa, Tari Turner

**Affiliations:** 1 Cochrane Australia, School of Public Health and Preventive Medicine, Monash University, 553 St Kilda Road, Melbourne, VIC 3004 Australia; 2 Department of Sexual and Reproductive Health and Research, World Health Organization, Geneva, Switzerland; 3 Department of Obstetrics and Gynecology, Faculty of Medical Sciences, University of Campinas, Campinas, SP Brazil

**Keywords:** Induction of labour, Decision-making, Guidelines, Guideline development, WHO-INTEGRATE, Evidence-to-decision framework, Health equity, World Health Organization, WHO

## Abstract

**Background:**

In 2019, WHO prioritized updating recommendations relating to three labour induction topics: labour induction at or beyond term, mechanical methods for labour induction, and outpatient labour induction. As part of this process, we aimed to review the evidence addressing factors beyond clinical effectiveness (values, human rights and sociocultural acceptability, health equity, and economic and feasibility considerations) to inform WHO Guideline Development Group decision-making using the WHO-INTEGRATE evidence-to-decision framework, and to reflect on how methods for identifying, synthesizing and integrating this evidence could be improved.

**Methods:**

We adapted the framework to consider the key criteria and sub-criteria relevant to our intervention. We searched for qualitative and other evidence across a variety of sources and mapped the eligible evidence to country income setting and perspective. Eligibility assessment and quality appraisal of qualitative evidence syntheses was undertaken using a two-step process informed by the ENTREQ statement. We adopted an iterative approach to interpret the evidence and provided both summary and detailed findings to the decision-makers. We also undertook a review to reflect on opportunities to improve the process of applying the framework and identifying the evidence.

**Results:**

Using the WHO-INTEGRATE framework allowed us to explore health rights and equity in a systematic and transparent way. We identified a lack of qualitative and other evidence from low- and middle-income settings and in populations that are most impacted by structural inequities or traditionally excluded from research. Our process review highlighted opportunities for future improvement, including adopting more systematic evidence mapping methods and working with social science researchers to strengthen theoretical understanding, methods and interpretation of the evidence.

**Conclusions:**

Using the WHO-INTEGRATE evidence-to-decision framework to inform decision-making in a global guideline for induction of labour, we identified both challenges and opportunities relating to the lack of evidence in populations and settings of need and interest; the theoretical approach informing the development and application of WHO-INTEGRATE; and interpretation of the evidence. We hope these insights will be useful for primary researchers as well as the evidence synthesis and health decision-making communities, and ultimately contribute to a reduction in health inequities.

**Supplementary Information:**

The online version contains supplementary material available at 10.1186/s12961-022-00901-7.

## Background

Induction of labour is an intervention undertaken in a healthcare setting that aims to “induce cervical ripening and/or to induce uterine contractions” [1, p. 6]. Pharmacological and mechanical methods may be used, either alone or in combination [1]. There are a wide range of obstetric, maternal and foetal indications for labour induction to improve maternal and neonatal health outcomes, including post-term pregnancy [2]. Rates of induction of labour are increasing across all income settings, with up to one in three babies now born after induction in some countries [3–6]. In 2011, WHO published 17 recommendations relating to the induction of labour [7]. In 2019, as part of a new “living guidelines” approach to WHO recommendations on maternal and perinatal health [8], the WHO Executive Guideline Steering Group prioritized the update of five recommendations relating to three labour induction topics where there was new, potentially important evidence:

- Induction of labour at or beyond term (developed 2011 and updated 2018 [9]),
- Mechanical methods for induction of labour (developed 2011), and
- Outpatient induction of labour (developed 2011).

The updates of these recommendations were published in October 2022, together with the evidence-to-decision frameworks [10–12].

### Developing the guidelines

As part of the United Nations Development Programme (UNDP)/UN Population Fund (UNFPA)/UN Children’s Fund (UNICEF)/WHO/World Bank Special Programme of Research, Development and Research Training in Human Reproduction guideline development process [13], evidence-to-decision (EtD) frameworks are drafted to inform the deliberations of the WHO Guideline Development Group (GDG) [14]. EtD frameworks allow for important factors relevant to health decision-making to be considered in a systematic and transparent way. Previous iterations of WHO induction of labour recommendations utilized the GRADE [Grading of Recommendations Assessment, Development and Evaluation] EtD framework for developing clinical practice guidelines. This widely accepted and used framework considers criteria of effectiveness, values, balance of benefits and harms, resources, equity, acceptability and feasibility [15].

**Fig. 1:**
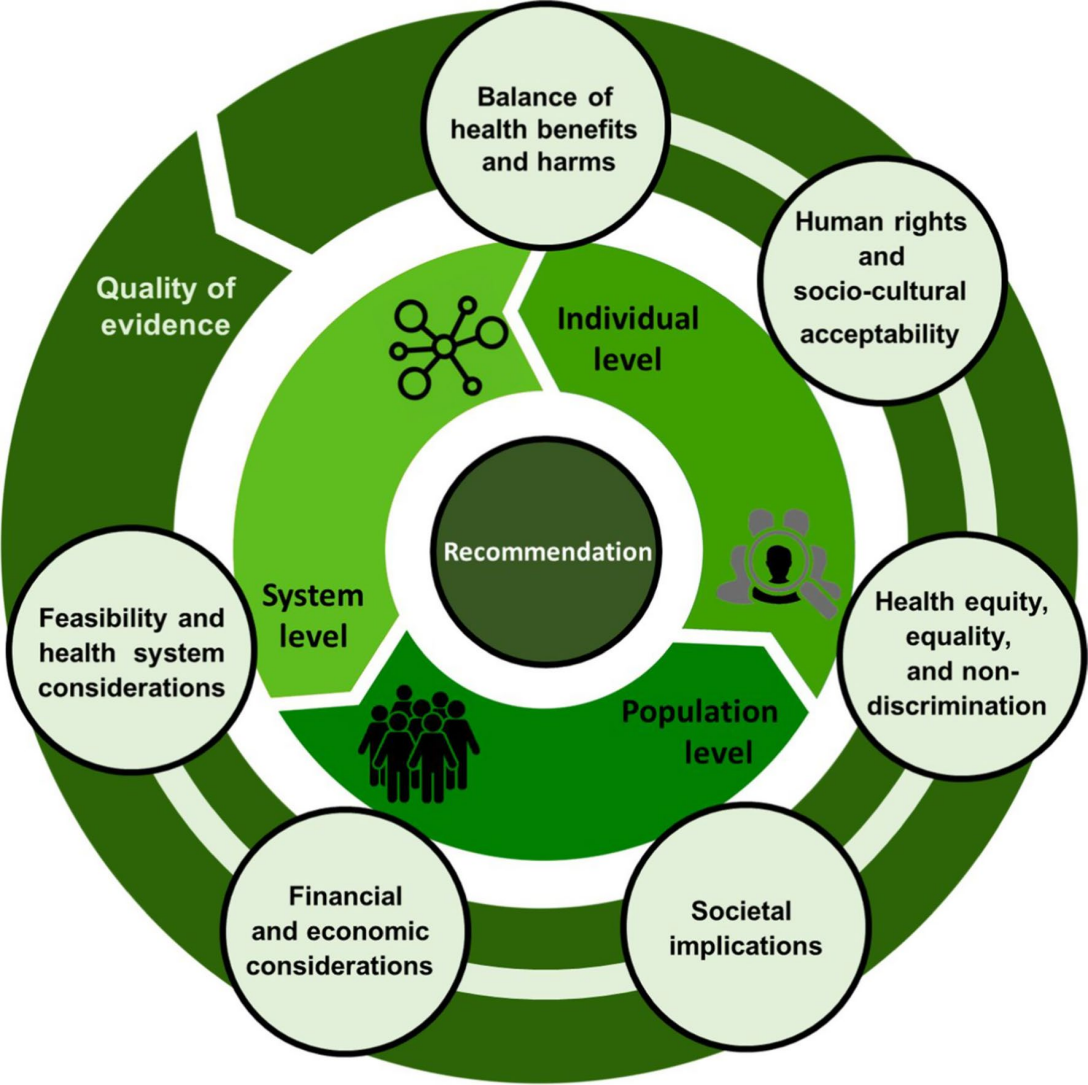
WHO-INTEGRATE framework, from Rehfuess 2019 [16]

For this update, we decided to use the WHO-INTEGRATE (INTEGRATe Evidence) framework for our work, as it enabled both a pragmatic approach given time and resource constraints, while ensuring the application of rigorous and comprehensive methods grounded in theory, and WHO norms and values. WHO-INTEGRATE also has a strong emphasis on and guidance around assessing equity and human (health) rights, which is critical for global recommendations with a primary intended audience of health decision-makers and clinicians in low- and middle-income settings [16]. WHO-INTEGRATE can be applied to individual-, population- and system-level interventions with varying degrees of complexity, and covers six criteria, and sub-criteria. Example questions are provided to help guide the collection of evidence (see Fig. 1 and section 7 of Additional file 1).

In this paper, we describe the methods used to apply WHO-INTEGRATE and present summary results of the evidence review for each of the EtD criteria for the three induction of labour topics. Detailed results of the evidence review are reported in section 1 of Additional file 1. We also reflect on our methods, process and evidence review outputs, discuss some of the limitations and challenges we encountered, gaps in the evidence base, and reflect on opportunities to improve the process of applying WHO-INTEGRATE.

## Methods

Cochrane’s Pregnancy and Childbirth Group reviewed the effectiveness evidence for the WHO GDG and prepared GRADE-based summary of findings tables and narrative summaries based on recent Cochrane review updates for each of the topics [1, 17, 18]. Two researchers from Cochrane Australia, Melissa Murano (Senior Research Officer) and Tari Turner (Associate Professor, Research), reviewed the evidence addressing factors beyond clinical effectiveness evidence using WHO-INTEGRATE to inform the decision-making of the 16-member GDG. Doris Chou of the WHO Department of Sexual and Reproductive Health and Research provided feedback throughout various stages of the evidence review.

### Application of WHO-INTEGRATE

We prepared a protocol for search, selection and assessment of evidence relevant to WHO-INTEGRATE criteria. Depending on the nature of the intervention under review, WHO-INTEGRATE authors suggest that criteria, sub-criteria and framing questions may be excluded from the assessment framework. The consideration of societal implications is of particular importance and relevance to complex interventions with several active components targeting a range of levels at a population or system level, multiple health and non-health outcomes, and long, complex causal pathways [16]. Induction of labour is not considered a complex intervention, and is an undertaken in a healthcare setting, where there are few, if any, concerns for sectors beyond health. We therefore decided at the outset that the criteria of societal implications (social and environmental impacts) were not relevant for our evidence assessment. As we undertook the analysis, additional sub-criteria and guiding questions were excluded. For example, we excluded the “Human rights and sociocultural acceptability” sub-criteria of “Impact on autonomy of concerned stakeholders” that assesses the extent to which an intervention may be justifiably imposed on individuals, communities or populations (e.g. restrictive public health orders during a pandemic) [19]. All framework adaptations are described in section 7 of Additional file 1.

We were aware that the GDG were not familiar with WHO-INTEGRATE as an EtD framework. To enable consistency of presentation and ease of understanding, we presented the results of the WHO-INTEGRATE assessment within the GRADE EtD heading structure. We provided a summary-level EtD document and a full report of the detailed findings for each EtD criteria ahead of the GDG meetings. We presented summarized versions of our findings for each criteria in slides during the GDG meeting for discussion. Mapping of the WHO-INTEGRATE and GRADE framework criteria and questions is documented in section 7 of Additional file 1.

### Qualitative evidence

#### Searches

We searched PubMed on 12 July 2021 and Epistemonikos on 13 July 2021 for systematic reviews and qualitative evidence syntheses (QES) of induction of labour published since 2018 (see Additional file 1 for search strategies). We restricted our search to these two sources since Epistemonikos is a database of systematic reviews relevant for health decision-making drawn from regularly updated searches of several key bibliographic databases, including Cochrane, PubMed, Embase, PsycINFO and CINAHL. The last searches for QES were conducted in 2018 for the update of WHO recommendations for induction of labour at or beyond term [9]. Therefore, our search dates were 01 Jan 2018 to 13 July 2021. Search strategies are documented in section 2 of Additional file 1. Reference lists of the three Cochrane reviews underpinning the recommendations being updated were also screened for eligible QES and primary qualitative studies [1, 17, 18], as was the reference list of the 2018 WHO update of recommendations for induction of labour at or beyond term [9].

#### Eligibility assessment and quality appraisal

Unlike accepted tools for assessing the quality of quantitative evidence syntheses (e.g. ROBIS [20], AMSTAR [A MeaSurement Tool to Assess systematic Reviews] 2 [21]), there are currently no validated widely accepted tools for appraising the quality of QES. Reporting guidelines for QES have been developed, notably the Enhancing transparency in reporting the synthesis of qualitative research (ENTREQ) statement [22] for QES more broadly, and meta-ethnography reporting guidance (eMERGe) [23] for meta-ethnographies.

We developed a two-step process for eligibility assessment and quality appraisal of QES informed primarily by the ENTREQ statement. Search results from PubMed and Epistemonikos were imported into Covidence [24] for deduplication and eligibility screening by a single researcher using five criteria. One researcher appraised eligible QES for quality using two additional criteria. The appraisals were then discussed with a second researcher to reach agreement on the quality rating. The Preferred Reporting Items for Systematic Reviews and Meta-Analyses (PRISMA) flow diagram [25] and QES eligibility and quality appraisal criteria are documented in section 3 of Additional file 1.

We aimed to identify a single, eligible QES of high quality to extract relevant findings from. If multiple eligible QES of high quality were identified, we selected the QES for inclusion based on the greatest breadth of included qualitative primary studies. All eligible QES were mapped to assess overlap of included studies and findings relevant to any of the three induction of labour topics and any of the EtD criteria. We also mapped the country income setting and perspectives (women and healthcare providers) of the included studies. Primary studies that were not included in the selected QES, but included in other high-quality QES, were then screened for additional relevant findings and to address gaps in the evidence in relation to country income setting or participant perspective (e.g. healthcare provider).

### Cost and cost-effectiveness evidence

Eligible primary cost-effectiveness studies were identified from a WHO living scoping review of cost-effectiveness evidence for maternal and perinatal health interventions [26]. Review, data extraction of detailed findings and quality appraisal using the extended Consensus on Health Economic Criteria (CHEC) list [27] was undertaken for trial-based economic studies by a single reviewer. A second reviewer confirmed the data extraction. We used three quality categories for the CHEC score (a maximum score of 19) as per van Eeden 2016 [28]: high (over 15), moderate (9–14) and low (< 8).

Model-based studies were not included in our analysis due to challenges in determining their risk of bias and transferability [29]. Detailed study characteristics of trial-based economic studies and brief characteristics of model-based economic studies are provided in section 1 of Additional file 1. Data extracted from trial-based economic studies are provided in Additional file 2.

### Other evidence and supporting information

To identify additional evidence and framing information for the criteria of equity, sociocultural acceptability (human rights) and feasibility, we screened the reference lists of the 2011 WHO recommendations for induction of labour [7] and the 2018 WHO update of recommendations for induction of labour at or beyond term [9].

### Preparation of EtD sections

The process of preparing the EtD sections was undertaken in a series of stages: data extraction, thematic coding and analysis. At each stage, the work was initially undertaken by a single researcher, discussed with and reviewed by a second researcher, and additional input gathered from a third researcher as needed. See section 6 of Additional file 1 for a more detailed description.

### Process review

As part of standard project close-out processes at Cochrane Australia, we reflected on the methods, processes and outputs domains of our work. We considered three open questions for each of these three domains: What worked well? What would we do differently next time? What have we learned for future work? These reflections were discussed within the team, and a summary of the key findings are presented in Table 2.

### Reflexivity statement

Melissa Murano has a social sciences and public health background, and has contributed to research to increase the uptake and implementation of evidence-informed policy and practice in low-income settings. Tari Turner is a mixed-methods researcher who has worked in high-, middle- and low-income settings, with a focus on systematic reviews of effectiveness evidence, and uptake and implementation of evidence-informed policy and practice. Both Melissa and Tari are white Australians living and working in a high-income setting. Throughout the evidence review, we reflected on our own social identities, views, values and beliefs and how these could influence our selection and interpretation of findings to minimize the possibility of bias. We are still learning how to do this well, and are aware that in many areas we may be blind to our privilege and biases.

The results of the evidence review were presented to and discussed with the WHO GDG, which has global/regional representation and includes people with a wide range of backgrounds, expertise views, and perspectives.

## Results

### Evidence review findings

Table 1 provides a summary of evidence review findings as presented to the GDG, together with WHO-INTEGRATE criteria/sub-criteria and framing questions. Findings that were considered by the GDG for all three induction of labour topics are presented first, followed by any findings relevant to a specific induction of labour topic. More detailed findings for each of the criteria is provided in section 1 of Additional file 1.

**Table 1:** Summary of evidence review findings aligned with GRADE EtD criteria

EtD criteria/sub-criteria	Main findings	Qualitative evidence sources
*Values*		
Sub-criteria Patients’/beneficiaries’ values in relation to health outcomes Framing questions To what extent do patients/beneficiaries value different health outcomes?	Labour induction in general Women placed great value on knowing about the potential benefits and harms of labour induction. They were concerned about the timing of delivery and its impact on the well-being of their baby (Will the baby be harmed by being born too early? Will the baby be harmed by being born too late?); the duration of the induction process until onset of labour and birth; the severity of pain; and the likelihood of caesarean delivery. Outpatient labour induction Women who underwent outpatient labour induction had additional safety concerns about going home, particularly in relation to being able to recognize if something was wrong. Some women were also concerned that labour might start suddenly at home. Women who were induced in an inpatient setting reported how painful induction was, whereas women who had outpatient induction did not mention pain.	[36, 40, 41, 43, 44]
*Resources*		
Sub-criteria Financial impact Framing questions What is the cost of the intervention? What is the overall budget impact of implementing the intervention? Do cost and budget impacts vary in the short- versus longer-term, and are they sustainable?	Economic evidence was very limited for the three induction of labour topics, derived from five trial-based primary studies conducted in high-income settings only. Economic analyses were eligible for all three induction of labour topics if the majority of the population was either at or beyond term and otherwise low-risk, and additionally for induction setting if the study compared the same induction method in both arms. Induction of labour at or beyond term Goeree et al. reported a mean cost saving per woman with labour induction of CAD 193 (95% CI 133 to 252) compared with expectant management [31], with effectiveness and resource utilization data from Hannah 1992 [45]. Grobman et al. reported that women who underwent labour induction used fewer resources in the antepartum period. During the intrapartum period, overall resource use was similar between the groups, however with different types of resources used. Postpartum resource use was largely similar between the two groups [32]. Resource utilization data were from a randomized controlled trial (RCT) conducted in 41 United States hospitals [46]. Outpatient induction of labour Adelson et al. undertook a cost analysis of outpatient vs inpatient labour induction [33], with effectiveness data from an RCT conducted in two Australian hospitals using vaginal PGE2 gel [47]. It is uncertain whether outpatient labour induction results in in-hospital cost savings or overall cost savings once the cost of the outpatient priming clinic was taken into account. Mechanical methods of induction of labour Ten Eikelder et al. undertook a cost-effectiveness analysis from a hospital perspective in the period from admission to antenatal care ward to discharge [34], with effectiveness data from an RCT in 29 hospitals in the Netherlands comparing oral misoprostol with Foley catheter [48]. It is unclear whether the Foley catheter is cost-saving for labour induction compared with oral misoprostol. The authors noted that at a willingness-to-pay threshold of at least €30,000 (2013 euro) per woman, oral misoprostol may be cost-effective for preventing caesarean section [34]. Van Baaren et al. undertook a cost-effectiveness analysis from a hospital perspective [35] using effectiveness data from an RCT in 12 hospitals in the Netherlands comparing Foley catheter with vaginal prostaglandin E2 gel [49]. It is unclear whether the Foley catheter is cost-saving for labour induction compared with vaginal PGE2 gel.	
Sub-criteria Ratio of costs and benefits Framing questions What is the value-for-money of the intervention, based on an appropriate choice of method, e.g. cost-effectiveness, cost–benefit or cost-utility?		
*Equity*		
Sub-criteria Impact on health equality and/or health equity Framing questions Is the intervention likely to reduce or increase existing health inequalities and/or health inequities? Does the intervention prioritize and/or aid those furthest behind? How do such impacts on health inequalities and /or health inequities vary over time, e.g. are initial increases likely to balance out over time, as interventions are scaled up?	We did not identify any direct evidence specific to induction of labour that addressed health equity. However, a 2015 WHO report on inequality in reproductive, maternal, newborn and child health states that “the poorest, the least educated and those residing in rural areas have lower health intervention coverage and worse health outcomes than the more advantaged” [50, p. xii]. The report also found that preventing and reducing morbidity and mortality in childbirth can play a key role in reducing overall health inequities [50]. Safe, effective and equitable implementation of labour induction for improved maternal and neonatal health outcomes could therefore potentially contribute to reducing inequities in maternal and perinatal health. Women living in low- to middle-income settings and/or remote or rural areas are less likely to have access to antenatal care to enable accurate gestational age estimation and risk assessment, or well-resourced facilities for monitoring and assessing maternal and foetal well-being during labour induction and/or performing caesarean sections if required [50–52]. This may reduce women’s ability to safely access labour induction, leading to poorer health outcomes, and reinforcing existing health inequities.	
Sub-criteria Distribution of benefits and harms of the intervention Framing questions How are the benefits and harms of the intervention distributed across the population? Who carries the burden (e.g. all), who benefits (e.g. a very small subgroup)?	The recently updated Cochrane reviews do not provide evidence to enable assessment of whether the balance of benefits and harms in relation to the three topics varies in different population subgroups [1, 17, 18].	
Sub-criteria Accessibility of intervention Framing questions How accessible—in terms of physical as well as informational access—is the intervention across different population groups?	The question of physical access was considered under the sub-criteria “Impact on health equality and/or health equity”. In terms of informational access, women from communities, populations and settings who are systematically denied equitable access to social, political and economic resources may experience greater barriers to participation in healthcare decision-making than indicated in the QES findings (see below under Acceptability) [53–55].	
*Acceptability and human rights*		
Sub-criteria Sociocultural acceptability of intervention to patients/beneficiaries and those implementing the intervention Framing questions Is the intervention socioculturally acceptable to patients/beneficiaries as well as to those implementing it? To which extent do patients/beneficiaries value different non-health outcomes?	Labour induction in general (women) Women have varying, and sometimes contradictory, views on the acceptability of labour induction. Acceptability varies according to women’s trust in their healthcare provider, their perception of birth as a natural process, their need for certainty, and the duration of waiting. Labour induction is widely acceptable to women when there is a recognized need to avert harm to the baby. Some women prefer interventions they can employ themselves to medical induction of labour. Outpatient induction of labour (women) Women value aspects of outpatient induction such as access to continuous social support; the freedom to move around and continue with their daily activities; being comfortable and being able to rest; having distractions waiting from waiting and pain. However, women who underwent outpatient induction also had additional safety concerns about going home. Coates et al. concluded that “outpatient IoL [induction of labour] is not preferable for all women, and individuals will have preferences about what constitutes a comfortable and safe environment for labour” [36, p. 26]. Labour induction in general (implementers) There is limited evidence available on the acceptability of labour induction to clinicians. A single study of obstetrician and midwife opinions on labour induction conducted in a high-income setting found that obstetricians felt there was a lack of clear evidence on the risks and benefits of labour induction to guide their decision-making. They were particularly concerned about neonatal safety and the potential for medical litigation, and were uncertain about the optimal timing for induction and the risks of caesarean birth following induction [42].	[36, 40–44]
Sub-criteria Sociocultural acceptability of intervention to the public and other relevant stakeholder groups Framing questions Is the intervention sensitive to sex, age, ethnicity, culture or language, sexual orientation or gender identity, disability status, education, socioeconomic status, place of residence or any other relevant characteristics?	We did not find any direct evidence to answer this question.	
Sub-criteria Accordance with universal human rights standards and principles Framing questions Is the intervention in accordance with universal human rights standards and principles?	Human rights include claims for health goods and services, and the right to information that enables women to be active agents when making decisions that affect their health [56]. Effective communication, respect and preservation of dignity, and emotional support are also vital to protection of human rights in healthcare [57]. Women generally wanted more timely and complete information about the risks and benefits, and process, of labour induction to enable them to make a competent, informed and voluntary decision about the intervention. They wanted to receive this information at a time and in a context that allowed them to process the information before a decision was required. There was a general perception that the timing and decision to induce labour was determined by the healthcare professional, or facility or system constraints. Women valued setting and systems that provided them with privacy, dignity and control, and where they could communicate their needs and have them responded to. Women were disturbed by the lack of privacy and freedom to move in the hospital setting. Women could also feel isolated and alone, and unable to access continuous support from induction through to birth. Some women experienced undergoing labour induction in hospital as a place of safety and security, knowing they had immediate access to healthcare providers and technology.	[36, 40, 41, 43, 44]
*Feasibility*		
Sub-criteria Need for, usage of and impact on infrastructure Framing questions How does the intervention interact with the need for and usage of the existing health system infrastructure (e.g. types of health facilities, health information system, medical products and technologies) at national and subnational levels? Is it likely to impact on these and their performance in positive or negative ways?	Labour induction is widely implemented in high-, middle- and low-income settings [41, 50–52], however performing induction of labour safely requires availability of appropriate drugs or mechanical devices, monitoring equipment and access to facilities for safe caesarean section. Inconsistent supply, or lack of, drugs and medical equipment and availability of appropriate facilities may be an issue in some settings.	
Sub-criteria Need for, usage of and impact on health workforce and human resources Framing questions How does the intervention interact with the need for and usage of the existing health workforce? Is it likely to impact on these in positive or negative ways, for example by affecting the number or distribution of staff, their skills, responsiveness or productivity?	Labour induction in general In lower- and middle-income country settings, trained healthcare worker shortages may reduce the feasibility of performing antenatal ultrasound scans and other risk assessment [50]. Time constraints can also be a barrier to information provision in antenatal care clinics [36]. Healthcare worker shortages in low- and middle-income country settings may require staff to attend to much higher numbers of women on the labour ward than in other settings. Providing the required level of support, assessment and monitoring in these settings may be challenging and impact on responsiveness [52]. Availability of surgical obstetric and operating theatre staff for women who require caesarean delivery if labour induction is not successful may also impact on feasibility. A higher number of induction deliveries are attended by medical doctors than non-induction deliveries [52]. This may have implications for the distribution and productivity of medical doctors, particularly in under-resourced settings Outpatient induction of labour There may be a greater need for staff who are available to answer questions, monitor and assess women remotely, and/or organize care or transfer for women who experience adverse reactions at home, such as uterine hyperstimulation, for outpatient induction of labour [18].	

Overall, our review was unable to identify qualitative evidence from geographical settings and populations most impacted by structural inequities and most often excluded from research. The included qualitative research from high-income settings did not explore the intersection of social categories and their impact on values and acceptability, rights and equity. The qualitative evidence relating to the period of care (intrapartum) supports the findings from other research in relation women’s experiences and human rights [30]. We did not identify any direct evidence specific to induction of labour that addressed health equity. Economic evidence was very limited for the three induction of labour topics, derived from five trial-based primary studies conducted in high-income settings only [31–35].

#### Qualitative study selection

A 2019 QES of women’s experiences of induction of labour (Coates 2019) [36] was selected for inclusion based on high quality and breadth of qualitative primary studies compared with two other eligible QES [37, 38] and one eligible scoping review [39]. Five primary qualitative studies providing additional data were identified and relevant findings extracted [40–44]. Characteristics of included studies and their rationale for inclusion can be found in section 8 of Additional file 1.

### Process review findings

Table 2 presents a summary of key findings from the process review that may be useful for researchers undertaking similar work. In future work, we would adopt more systematic evidence mapping methods, consider additional relevant frameworks, and work with social science researchers to strengthen our theoretical understanding, methods and interpretation of the evidence. We learnt that considering the inclusion, reporting and consideration of diverse populations and settings in the primary qualitative evidence base before extracting and interpreting findings can inform considerations around equity. Providing the GDG with an evidence gap map for key settings, populations and characteristics of interest would further enhance discussion of equity, provide a research agenda, and focus future recommendation update work on overlooked settings and populations.

**Table 2:** Key process review findings

What worked well?	What would we do differently?	What have we learned for future work?
Methods		
Including WHO and UN statements and reports allowed us to identify key background information to frame and focus our qualitative findings. Iteratively considering the WHO-INTEGRATE criteria, sub-criteria and framing questions as we became more familiar with WHO-INTEGRATE and the body of evidence resulted in extracting findings most relevant to our decision-making context. Restricting selection of economic evidence to trial-based studies, to avoid challenges with assessing model validity and generalizability. Adapting our a priori protocol to include additional primary studies beyond the included QES that addressed evidence gaps. This allowed us to include a study on healthcare provider attitudes to induction of labour [42], the only study conducted in a low-income setting [40], and studies with additional findings relating to pain and women experiencing prolonged pregnancy [41, 43, 44].	Searches Investigate usefulness of separate search strategies and/or additional search sources for areas with limited evidence coverage e.g. feasibility and/or equity. Search for primary qualitative studies with important perspectives, settings, populations or subgroups published after the last search dates of the included QES. This was done informally, but the process would have benefited from a more systematic approach. Mapping the evidence (gaps) Use systematic mapping methods to identify evidence gaps for each of the WHO-INTEGRATE criteria in settings and populations of specific interest (e.g. country income setting, populations who are systematically discriminated against and denied equitable access to social, political and economic resources). This would both emphasize these issues for discussion, and guide evidence searches for future recommendation updates. Additional frameworks Explicitly consider intersectional theory and approaches when designing methods to search for, identify and interpret qualitative findings. This may allow greater coherence and credibility of evidence presentation. Including frameworks relating to the period and/or process of care may also allow findings to be differentiated that are specific to the intervention being examined or more broadly applicable to the period and/or process of care.	The literature on feasibility and equity is sparse (and possibly not current) if relying on QES and screening reference lists of WHO recommendations and Cochrane reviews. Separate searches for additional information are likely to be required. Further development of methods for search, identification and interpretation of evidence would result in more robust analysis of the intersection of all factors impacting equity. Inclusion of a social sciences equity researcher on the team would strengthen theoretical understanding, application of methods and interpretation of evidence. Production of evidence gap maps may help highlight which settings and populations are not included in the evidence base, providing a research agenda for primary qualitative research and a baseline for targeted searching in recommendation updates.
Process		
Iterative collaboration between team members to ensure WHO-INTEGRATE was applied consistently, and to identify relevant findings that represented breadth and diversity of voices and experiences.	Extract population/setting characteristics of QES included studies and any additional primary studies before extracting and interpreting findings to better inform discussions around inequity. Discuss the extent to which prior knowledge of the effectiveness evidence is likely to influence the team’s interpretation of the qualitative and other evidence findings and explicitly take this into account during the process to reduce bias.	Understanding the included and excluded settings and populations in the qualitative evidence base provides vital information to consider questions of inequity.
Outputs		
Providing the GDG with a high-level evidence review summary in the EtD documents and a supplemental file with more detailed findings.	Provide three outputs to guide decision-making and future recommendation updates: (1) high-level summary of evidence review for each criterion in EtD document; (2) detailed findings from evidence review for each criterion in a supplemental file, including methods and characteristics of studies; and (3) evidence gap map for each criterion, including country income setting and other pre-determined characteristics of interest. Investigate how best to overcome the challenges posed by the lack of qualitative evidence from low- and middle-income country settings for many key EtD domains.	Using WHO-INTEGRATE and providing both high-level and detailed findings to the GDG centred women’s voices and allowed us to explore health rights and inequity in detail. This facilitated meaningful consideration of women’s experiences, values and preferences, impacts on rights and inequity, as well as feasibility issues, particularly in low- and middle-income settings.

## Discussion

### The WHO-INTEGRATE framework

Using WHO-INTEGRATE and providing both high-level and detailed evidence review findings to the GDG centred women’s voices and allowed us to explore health rights and inequity in a detailed, systematic and transparent way within the constraints of time and resources. This facilitated more meaningful consideration of the interplay of women’s experiences, values and preferences, and socio-structural impacts on feasibility, rights and equity.

WHO-INTEGRATE assists in the detailed consideration of these issues by clearly identifying the important criteria and sub-criteria to evaluate sociocultural acceptability, rights and equity, and providing framing questions to guide the selection and assessment of a variety of evidence sources. This detailed approach is particularly valuable in helping researchers who do not have social sciences expertise to identify and reflect more deeply on the upstream structural issues that are the root causes of social and economic determinants of health [58]. Retaining a tight focus on equity in relation to differential risk and/or outcomes risks rendering the interplay of structural racism, gender and heteronormative bias, ableism and ongoing impacts of colonization invisible and therefore obscuring accountability and potential solutions [58–60]. Using WHO-INTEGRATE to highlight issues of equity and differential impacts on rights, acceptability, resourcing and feasibility in published EtDs may also assist guideline users in adapting global guidelines to their local context to achieve higher relevance and acceptability, more targeted implementation, and ultimately better health outcomes [61, 62].

Adequately assessing rights and equity is challenging. Equity and rights are often poorly dealt with in EtDs for guidelines, resulting in a narrow or limited approach that does not consider these critical issues through the entire EtD process [63, 64]. This may be due to a combination of lack of evidence, lack of time and resources, lack of training [65] or limitations posed by researcher subjectivity [60]. When equity is considered, it is mostly in relation to baseline risks and differential outcomes in the quantitative effectiveness evidence (generally through the lens of place of residence), with little use of qualitative or other evidence sources. The linear application of EtD criteria also overlooks the overlapping nature of equity and rights considerations, and their intersection with values, acceptability, resources and feasibility [63].

There may be opportunity for the WHO-INTEGRATE developers to incorporate a more matrix-like, intersectional approach to assist EtD developers in considering multiple forms of power, privilege, inequality and identity simultaneously, rather than adopting a single-issue, additive or sequential approach [66]. This would serve to highlight the underlying power structures both within and between countries that lead to differential experiences of and access to healthcare and health outcomes, avoiding more individualistic or even pathologizing interpretations [66, 67].

Other users of WHO-INTEGRATE have also noted the overlapping and intersecting nature of the criteria, whereby values, acceptability, rights, equity, resources and feasibility must be considered in an iterative manner [68]. Additionally, separating out the question of human/health rights into a separate criterion (rather than considering this as a sub-criterion under sociocultural acceptability) and/or incorporating consideration of rights into the equity criteria could strengthen the analytical approach, given that rights may be differentially upheld or withdrawn based on categories of social, economic and political inequality.

The use of additional frameworks developed specifically for the healthcare period (e.g. WHO Quality of Care Framework for Maternal and Newborn Health [57]) or process (e.g. shared decision-making), may also provide a more systematic and targeted analysis of qualitative and other evidence to guide and inform panel discussions. Interdisciplinary collaboration with social science researchers working in the equity field could also strengthen theoretical understanding and application, methods and analytical approaches [60].

### The evidence

Our review identified a lack of qualitative and other evidence from low- and middle-income settings and in populations that are most impacted by structural inequities and/or traditionally excluded from research. The qualitative research undertaken in high-income settings either explicitly excluded women from diverse populations on the basis of language who may have different experiences, values and expectations of accessing maternal healthcare, or did not identify, report or explore the intersection of social categories and their impact on values and acceptability, rights and equity.

The lack of primary qualitative research undertaken in settings and populations of highest need is a common challenge faced by those synthesizing evidence for healthcare decision-making [65]. The qualitative evidence for our review was limited to a subset of women from high-income settings who were mostly white and tended to be well educated (see section 9 in Additional file 1 for primary study participant characteristics and reporting). This leads to concerns about lack of generalizability and uncertainty in the value placed on health and non-health outcomes by women with differing abilities and/or from diverse populations, and women in other country income settings [65]. As reviewers, we had to draw on broader WHO reports of maternal care to interpret the qualitative evidence and address issues of equity and [health] rights from our positionality as white researchers in a high-income setting, which may further limit the usefulness of our review for decision-makers and users in other contexts.

Our experience highlighted an important role for evidence gap maps [69–71] in making explicit the need for primary research in low- and middle-income settings and in populations who are systematically denied access to social, economic and political power; making a case for reducing “research waste” in settings and populations that have been adequately researched to answer the question(s) of interest; and focusing future guideline update resources on filling identified priority gaps, rather than undertaking further evidence evaluation of well- or over-researched and understood settings and populations.

In the absence of qualitative evidence from low- and middle-income settings, it is even more important to maximize the usefulness of the quantitative evidence for evaluation of impacts on equity. This can be done by undertaking subgroup analyses considering relative and absolute effects based on key indicators, such as those proposed by PROGRESS-Plus, if the primary studies present disaggregated data [72]. At a minimum, undertaking a subgroup analysis based on country income level can indicate whether there is any important variation in health outcomes for the intervention of interest to inform consideration of resources, equity and feasibility [65]. While we were constrained by scope and timeline in our review, considering prevalence and other epidemiological information on induction of labour may also have helped highlight structural issues, including system and clinician biases that impact on equitable access when considered together with the qualitative and other evidence [73].

In summary, we identified significant gaps in the qualitative and other evidence for assessing values, acceptability, rights, equity, resource requirements and feasibility in populations and settings of need and interest. In spite of this lack of direct evidence, WHO-INTEGRATE enabled us to consider questions of acceptability, rights and equity in a systematic and transparent way. However, we also found that the linear application of discrete criteria limited our ability to consider the interaction of key EtD domains and the resulting impact on rights and equity.

There are a few limitations on this work, largely arising from limited time and resources. For example, searches for QES were only undertaken in two databases; we did not systematically search for additional relevant primary qualitative studies published after the selected QES search date; and duplicate review was not used for some elements of the process. Having a team member with health economics expertise would also have strengthened our ability to integrate this evidence. However, we believe that the methods used are robust, reflecting the practical nature of the undertaking and potentially increasing generalizability to similar real-world activities. A number of issues were identified that require addressing, or would have strengthened our work: the generation of primary qualitative evidence in settings and populations that are of high need and/or overlooked; incorporating an integrative intersectional approach into the theoretical underpinnings and application of WHO-INTEGRATE; the benefit of interdisciplinary collaboration with social scientists to adequately address rights and equity; and a need to continue addressing barriers to participation so that researchers from low- and middle-income countries can lead this important work. In addressing these needs, evaluation of non-effectiveness evidence for health policy decision-making can advocate for change by highlighting structures that uphold both privilege and discrimination, and draw attention to forms of evidence and experiences from individuals, communities and populations who are often excluded from the decision-making process.

## Conclusion

Using the WHO-INTEGRATE EtD framework, we undertook an evidence review of key criteria to inform health policy decision-making in a global guideline for induction of labour. During a reflective process, we identified both challenges and opportunities relating to the lack of evidence in populations and settings of need and interest, the theoretical approach informing the development and application of WHO-INTEGRATE, and interpretation of the evidence. We hope these insights will be useful for individual researchers as well as the evidence generation and health policy decision-making communities, and ultimately contribute to a reduction in health inequities.

## Supplementary Information

**Additional file 1. **Supplementary methods and data for “Using the WHO-INTEGRATE evidence-to-decision framework in the development of induction of labour recommendations”. Detailed evidence review findings, 2. Search strategies, 3. Qualitative evidence eligibility and quality assessment, 4. Qualitative evidence selection, 5. Cost and cost-effectiveness evidence, 6. EtD framework mapping, 7. Characteristics of included qualitative studies, 8. Participant characteristics in included qualitative studies, 9. References.

**Additional file 2. **Economic data extraction “Using the WHO-INTEGRATE evidence-to-decision framework in the development of induction of labour recommendations”. 1. Economic study design and characteristics, 2. Treatment and cost data.

## Data Availability

Provided in Additional files 1 and 2.

## Abbreviations

AMSTAR: A MeaSurement Tool to Assess systematic Reviews
CHEC: Consensus on Health Economic Criteria
EtD: Evidence-to-decision
eMERGe: Meta-ethnography reporting guidance
ENTREQ: Enhancing transparency in reporting the synthesis of qualitative research
GDG: WHO Guideline Development Group
GRADE: Grading of Recommendations Assessment, Development and Evaluation
IOL: Induction of labour
PRISMA: Preferred Reporting Items for Systematic Reviews and Meta-Analyses
QES: Qualitative evidence syntheses
RCT: Randomized controlled trial
WHO-INTEGRATE: INTEGRATe Evidence framework
UN: United Nations

## References

[1] Middleton P, Shepherd E, Morris J, Crowther CA, Gomersall JC (2020). Induction of labour at or beyond 37 weeks' gestation. Cochrane Database Syst Rev.

[2] Berghella V, Bellussi F, Schoen CN (2020). Evidence-based labor management: induction of labor (part 2). Am J Obstetrics Gynecol MFM.

[3] Seijmonsbergen-Schermers AE, van den Akker T, Rydahl E, Beeckman K, Bogaerts A, Binfa L (2020). Variations in use of childbirth interventions in 13 high-income countries: a multinational cross-sectional study. PLoS Med.

[4] Australian Institute of Health Welfare. Australia's mothers and babies. Canberra: AIHW. 2021. https://www.aihw.gov.au/reports/mothers-babies/australias-mothers-babies. Accessed 01 Dec 2021.

[5] Vogel JP, Souza JP, Gülmezoglu AM (2013). Patterns and outcomes of induction of labour in Africa and Asia: a secondary analysis of the WHO Global Survey on Maternal and Neonatal Health. PLoS ONE.

[6] Guerra GV, Cecatti JG, Souza JP, Faúndes A, Gülmezoglu AM, Passini R (2011). Elective induction versus spontaneous labour in Latin America. Bull World Health Organ.

[7] WHO recommendations for induction of labour. Geneva: World Health Organization. 2011. 23586118

[8] Vogel JP, Dowswell T, Lewin S, Bonet M, Hampson L, Kellie F (2019). Developing and applying a 'living guidelines' approach to WHO recommendations on maternal and perinatal health. BMJ Glob Health.

[9] WHO recommendations: Induction of labour at or beyond term. Geneva: World Health Organization. https://apps.who.int/iris/bitstream/handle/10665/277233/9789241550413-eng.pdf?ua=1; 2018. 30629393

[10] WHO recommendations on induction of labour, at or beyond term. Geneva: World Health Organization. https://www.who.int/publications/i/item/9789240052796; 2022 36279382

[11] WHO recommendations on mechanical methods for induction of labour. Geneva: World Health Organization. https://www.who.int/publications/i/item/9789240055780; 2022 36279383

[12] WHO recommendations on outpatient settings for induction of labour. Geneva: World Health Organization. https://www.who.int/publications/i/item/9789240055810; 2022 36279384

[13] WHO/HRP: Sexual and Reproductive Health and Research (SRH): Guidelines development. World Health Organization. https://www.who.int/teams/sexual-and-reproductive-health-and-research-(srh)/guidelines; 2022. Accessed 8 Apr 2022.

[14] World Health Organization. WHO handbook for guideline development. 2nd ed: World Health Organization; 2014. https://apps.who.int/iris/handle/10665/145714.

[15] Alonso-Coello P, Oxman AD, Moberg J, Brignardello-Petersen R, Akl EA, Davoli M (2016). GRADE Evidence to Decision (EtD) frameworks: a systematic and transparent approach to making well informed healthcare choices. 2: Clinical practice guidelines. BMJ.

[16] Rehfuess EA, Stratil JM, Scheel IB, Portela A, Norris SL, Baltussen R (2019). The WHO-INTEGRATE evidence to decision framework version 10: integrating WHO norms and values and a complexity perspective. BMJ Glob Health.

[17] de Vaan MDT, ten Eikelder MLG, Jozwiak M, Palmer KR (2019). Mechanical methods for induction of labour. Cochrane Database of Syst Rev.

[18] Alfirevic Z, Gyte GML, Nogueira Pileggi V, Plachcinski R, Osoti AO, Finucane EM (2020). Home versus inpatient induction of labour for improving birth outcomes. Cochrane Database of Syst Rev.

[19] Public health: ethical issues. London, UK: The Nuffield Council on Bioethics. 2007. https://www.nuffieldbioethics.org/publications/public-health.

[20] Whiting P, Savović J, Higgins JPT, Caldwell DM, Reeves BC, Shea B (2016). ROBIS: a new tool to assess risk of bias in systematic reviews was developed. J Clin Epidemiol.

[21] Shea BJ, Reeves BC, Wells G (2017). AMSTAR 2: a critical appraisal tool for systematic reviews that include randomised or non-randomised studies of healthcare interventions, or both. BMJ.

[22] Tong A, Flemming K, McInnes E, Oliver S, Craig J (2012). Enhancing transparency in reporting the synthesis of qualitative research: ENTREQ. BMC Med Res Methodol.

[23] France EF, Cunningham M, Ring N, Uny I, Duncan EA, Jepson RG (2019). Improving reporting of meta-ethnography: the eMERGe reporting guidance. BMC Med Res Methodol.

[24] Covidence systematic review software. Melbourne, Australia: Veritas Health Innovation;(2021)

[25] Page MJ, McKenzie JE, Bossuyt PM, Boutron I, Hoffmann TC, Mulrow CD (2021). The PRISMA 2020 statement: an updated guideline for reporting systematic reviews. BMJ.

[26] Eddy K, Vogel J. Cost-effectiveness evidence for maternal and perinatal health interventions: living scoping review. World Health Organization 2020. http://osf.io/jwtge. Accessed 5 Jul 2021

[27] Evers S, Goossens M, De Vet H, Van Tulder M, Ament A (2005). Criteria list for assessment of methodological quality of economic evaluations: Consensus on Health Economic Criteria. Int J Technol Assess Health Care.

[28] van Eeden M, van Heugten CM, van Mastrigt GA, Evers SM (2016). Economic evaluation of studies of self-management interventions in chronic diseases: a systematic review. Int J Technol Assess Health Care.

[29] Wijnen BFM, Van Mastrigt G, Redekop WK, Majoie HJM, De Kinderen RJA, Evers S (2016). How to prepare a systematic review of economic evaluations for informing evidence-based healthcare decisions: data extraction, risk of bias, and transferability (part 3/3). Expert Rev Pharmacoecon Outcomes Res.

[30] Downe S, Finlayson K, Oladapo OT, Bonet M, Gülmezoglu AM (2018). What matters to women during childbirth: a systematic qualitative review. PLoS ONE.

[31] Goeree R, Hannah M, Hewson S (1995). Cost-effectiveness of induction of labour versus serial antenatal monitoring in the Canadian Multicentre Postterm Pregnancy Trial. CMAJ Can Med Assoc J.

[32] Grobman WA, Sandoval G, Reddy UM, Tita ATN, Silver RM (2020). Health resource utilization of labor induction versus expectant management. Am J Obstet Gynecol.

[33] Adelson PL, Wedlock GR, Wilkinson CS, Howard K, Bryce RL, Turnbull DA (2013). A cost analysis of inpatient compared with outpatient prostaglandin E2 cervical priming for induction of labour: results from the OPRA trial. Aust Health Rev.

[34] ten Eikelder M, van Baaren GJ, Oude Rengerink K, Jozwiak M, de Leeuw JW, Kleiverda G (2018). Comparing induction of labour with oral misoprostol or Foley catheter at term: cost-effectiveness analysis of a randomised controlled multi-centre non-inferiority trial. BJOG.

[35] van Baaren GJ, Jozwiak M, Opmeer BC, Oude Rengerink K, Benthem M, Dijksterhuis MGK (2013). Cost-effectiveness of induction of labour at term with a Foley catheter compared to vaginal prostaglandin E2 gel (PROBAAT trial). BJOG.

[36] Coates R, Cupples G, Scamell A, McCourt C (2019). Women's experiences of induction of labour: qualitative systematic review and thematic synthesis. Midwifery.

[37] Akuamoah-Boateng J, Spencer R (2018). Woman-centered care: Women's experiences and perceptions of induction of labor for uncomplicated post-term pregnancy: a systematic review of qualitative evidence. Midwifery.

[38] Lou S, Hvidman L, Uldbjerg N, Neumann L, Jensen TF, Haben JG (2019). Women's experiences of postterm induction of labor: a systematic review of qualitative studies. Birth.

[39] Coates D, Goodfellow A, Sinclair L (2020). Induction of labour: Experiences of care and decision-making of women and clinicians. Women Birth.

[40] Ezeanochie M, Olagbuji B, Ande A (2013). Women's concerns and satisfaction with induced labour at term in a Nigerian population. Niger Postgrad Med J.

[41] Henderson J, Redshaw M (2013). Women's experience of induction of labor: a mixed methods study. Acta Obstet Gynecol Scand.

[42] Nippita TA, Porter M, Seeho SK, Morris JM, Roberts CL (2017). Variation in clinical decision-making for induction of labour: a qualitative study. BMC Pregnancy Childbirth.

[43] Wessberg A, Lundgren I, Elden H (2017). Being in limbo: Women’s lived experiences of pregnancy at 41 weeks of gestation and beyond—a phenomenological study. BMC Pregnancy Childbirth.

[44] Westfall RE, Benoit C (2004). The rhetoric of "natural" in natural childbirth: childbearing women's perspectives on prolonged pregnancy and induction of labour. Soc Sci Med.

[45] Hannah ME, Hannah WJ, Hellmann J, Hewson S, Milner R, Willan A (1992). Induction of labor as compared with serial antenatal monitoring in post-term pregnancy: a randomized controlled trial. N Engl J Med.

[46] Grobman WA, Rice MM, Reddy UM, Tita AT, Silver RM, Mallett G (2018). Labor induction versus expectant management in low-risk nulliparous women. N Engl J Med.

[47] Wilkinson C, Bryce R, Adelson P, Turnbull D (2015). A randomised controlled trial of outpatient compared with inpatient cervical ripening with prostaglandin E_2_ (OPRA study). BJOG.

[48] ten Eikelder ML, Rengerink KO, Jozwiak M, De Leeuw JW, De Graaf IM, Van Pampus MG (2016). Induction of labour at term with oral misoprostol versus a Foley catheter (PROBAAT-II): a multicentre randomised controlled non-inferiority trial. Lancet.

[49] Jozwiak M, Rengerink KO, Benthem M, Van Beek E, Dijksterhuis MG, De Graaf IM (2011). Foley catheter versus vaginal prostaglandin E2 gel for induction of labour at term (PROBAAT trial): an open-label, randomised controlled trial. The Lancet.

[50] State of inequality (2015). Reproductive, maternal, newborn and child health.

[51] WHO recommendations on antenatal care for a positive pregnancy experience. Geneva, CH: World Health Organization. 2016; (9241549912). 28079998

[52] Vogel JP, Gülmezoglu AMM, Hofmeyr GJ, Temmerman M (2014). Global perspectives on elective induction of labor. Clin Obstet Gynecol.

[53] Protheroe J, Brooks H, Chew-Graham C, Gardner C, Rogers A (2013). ‘Permission to participate?’ A qualitative study of participation in patients from differing socio-economic backgrounds. J Health Psychol.

[54] Joseph-Williams N, Elwyn G, Edwards A (2014). Knowledge is not power for patients: a systematic review and thematic synthesis of patient-reported barriers and facilitators to shared decision making. Patient Educ Couns.

[55] Primary health care on the road to universal health coverage: 2019 global monitoring report. Geneva, CH: World Health Organization. 2021; (9240004270).

[56] Technical guidance on the application of a human-rights based approach to the implementation of policies and programmes to reduce preventable maternal morbidity and mortality. United Nations Human Rights Council New York. 2012. https://www.ohchr.org/Documents/HRBodies/HRCouncil/RegularSession/Session21/A-HRC-21-22_en.pdf. Accessed 23 Aug 2021.

[57] Tunçalp Ӧ, Were W, MacLennan C, Oladapo O, Gülmezoglu A, Bahl R (2015). Quality of care for pregnant women and newborns—the WHO vision. BJOG.

[58] Schram A, Townsend B, Mackean T, Freeman T, Fisher M, Harris P (2022). Promoting action on structural drivers of health inequity: principles for policy evaluation. Evid Policy.

[59] Eneanya ND, Boulware L, Tsai J, Bruce MA, Ford CL, Harris C, et al. Health inequities and the inappropriate use of race in nephrology. Nature Reviews Nephrology 2021:1–11. 10.1038/s41581-021-00501-8 PMC8574929 34750551

[60] Lett E, Adekunle D, McMurray P, Asabor EN, Irie W, Simon MA (2022). Health equity tourism: ravaging the justice landscape. J Med Syst.

[61] Wang Z, Grundy Q, Parker L, Bero L (2019). Health promoter, advocate, legitimizer—the many roles of WHO guidelines: a qualitative study. Health Res Policy Syst.

[62] Maaløe N, Ørtved AMR, Sørensen JB, Sequeira Dmello B, van den Akker T, Kujabi ML (2021). The injustice of unfit clinical practice guidelines in low-resource realities. Lancet Glob Health.

[63] Dewidar O, Tsang P, León-García M, Mathew C, Antequera A, Baldeh T (2020). Over half of the WHO guidelines published from 2014 to 2019 explicitly considered health equity issues: a cross-sectional survey. J Clin Epidemiol.

[64] Barnabe C, Pianarosa E, Hazlewood G (2021). Informing the GRADE evidence to decision process with health equity considerations: demonstration from the Canadian rheumatoid arthritis care context. J Clin Epidemiol.

[65] Pottie K, Welch V, Morton R, Akl EA, Eslava-Schmalbach JH, Katikireddi V (2017). GRADE equity guidelines 4: considering health equity in GRADE guideline development: evidence to decision process. J Clin Epidemiol.

[66] May VM (2015). Pursuing intersectionality, unsettling dominant imaginaries.

[67] Crenshaw KW (2017). On intersectionality: essential writings.

[68] Stratil JM, Paudel D, Setty KE, Menezes de Rezende CE, Monroe AA, Osuret J, et al. Advancing the WHO-INTEGRATE Framework as a Tool for Evidence-Informed, Deliberative Decision-Making Processes: Exploring the Views of Developers and Users of WHO Guidelines. Int J Health Policy Manag.10.34172/ijhpm.2020.193. 10.34172/ijhpm.2020.193 PMC9309924 33131223

[69] Saran A. Developing evidence maps to identify equity issues that could inform the design of a complex public health review. London, UK: Cochrane. https://www.youtube.com/watch?v=Pqq9U57DZVQ; 2021. Accessed 14 Feb 2022.

[70] Saran A, White H (2018). Evidence and gap maps: a comparison of different approaches. Campbell Syst Rev.

[71] Snilstveit B, Bhatia R, Rankin K, Leach B. 3ie evidence gap maps. a starting point for strategic evidence production and use. New Delhi: International Initiative for Impact Evaluation (3ie) 2017.

[72] Welch V, Dewidar O, Tanjong Ghogomu E, Abdisalam S, Al Ameer A, Barbeau VI (2022). How effects on health equity are assessed in systematic reviews of interventions. Cochrane Database Syst Rev.

[73] Welch VA, Akl EA, Guyatt G, Pottie K, Eslava-Schmalbach J, Ansari MT (2017). GRADE equity guidelines 1: considering health equity in GRADE guideline development: introduction and rationale. J Clin Epidemiol.

