# Supplementary material for: Using the WHO-INTEGRATE evidence-to-decision framework to develop recommendations for induction of labour
Source: Health Res Policy Syst. 2022 Nov 7;20:125. doi: 10.1186/s12961-022-00901-7 (PMC9641799; doi:10.1186/s12961-022-00901-7)
Supplement: Supplementary file 1 — Additional file 1. Supplementary methods and data for “Using the WHO-INTEGRATE evidence-to-decision framework in the development of induction of labour recommendations”. Detailed evidence review findings, 2. Search strategies, 3. Qualitative evidence eligibility and quality assessment, 4. Qualitative evidence selection, 5. Cost and cost-effectiveness evidence, 6. EtD framework mapping, 7. Characteristics of included qualitative studies, 8. Participant characteristics in included qualitative studies, 9. References. [file 12961_2022_901_MOESM1_ESM.docx]

Additional file 1: Supplementary methods and data for ‘Using the WHO-INTEGRATE evidence-to-decision framework in the development of induction of labour recommendations’.

Contents

[1. Evidence review: detailed findings 2](#_Toc106176008)

[Values 2](#_Toc106176009)

[Resources 3](#_Toc106176010)

[Equity 7](#_Toc106176011)

[Acceptability & human rights 9](#_Toc106176012)

[Feasibility 12](#_Toc106176013)

[2. Search strategies 15](#_Toc106176014)

[PubMed 15](#_Toc106176015)

[Epistemonikos 15](#_Toc106176016)

[3. Qualitative evidence eligibility and quality assessment 15](#_Toc106176017)

[Eligibility criteria 15](#_Toc106176018)

[Quality criteria 16](#_Toc106176019)

[Domain judgements 16](#_Toc106176020)

[4. Qualitative evidence selection 16](#_Toc106176021)

[5. Cost and cost-effectiveness evidence 16](#_Toc106176022)

[6. Preparation of EtD sections 17](#_Toc106176023)

[7. EtD Framework mapping 18](#_Toc106176024)

[8. Characteristics of included qualitative studies 23](#_Toc106176025)

[9. Participant characteristics in included qualitative studies 24](#_Toc106176026)

[10. References 26](#_Toc106176027)

# Evidence review: detailed findings

Detailed findings common to all three induction of labour topics (timing, setting, mechanical methods) for each EtD criteria are provided below. Any additional findings specific to a topic are noted separately. A high-level summary for each EtD criteria is provided in each induction of labour topic EtD document.

## Values

### Framing questions

- Is there important uncertainty about or variability in how much people value the main outcomes? (GRADE)
- To what extent do patients/beneficiaries value different health outcomes? (INTEGRATE)
- How important are the health outcomes linked with an intervention to the people affected?
- Which outcomes matter most?
- How do they feel about the possible benefits vs the possible harms of labour induction? (WHO GL Handbook)

### Labour induction in general

Key outcomes of interest identified in the QES and additional qualitative studies of women’s experiences of induction of labour and post-term pregnancy are:

- timing of delivery and its impact on the wellbeing of their baby (will the baby be harmed by being born too early? will the baby be harmed by being born too late?)
- duration of the induction process until onset of labour and delivery
- severity of pain
- likelihood of caesarean delivery.

In the Coates 2019 QES, women’s desire for the safety of their baby outweighed their concerns about labour induction, however women also expressed fear that induction would cause their baby to be born before it was ready. Women wanted more information about the potential benefits and harms of labour induction, particularly in relation to their individual circumstances [1].

An additional study of women who were at 41+ weeks in their pregnancy found that some women trusted that their baby was well and would arrive when it was ready. While women experienced feelings of frustration and irritation that labour had not started, they understood that being overdue was normal. Other women felt less secure in their body’s ability to give birth, and this feeling could be exacerbated by opinions and advice from others. Worry that there was something wrong with their baby increased over time [2].

An additional study conducted in a lower-middle income setting found that the majority of women were satisfied with the induction process. The most common reasons given for dissatisfaction with labour induction were that it resulted in a caesarean delivery, painful labour, and the time between induction and delivery was too long [3].

In the Coates 2019 QES, women whose induction resulted in a caesarean section reported being disappointed and sad [1].

### Outpatient labour induction

In the Coates 2019 QES, women who underwent outpatient labour induction had additional safety concerns about going home, particularly in relation to being able to recognise if something was wrong. Some women were also concerned that labour might start suddenly at home. Women who were induced in an inpatient setting reported how painful induction was, whereas women who had outpatient induction did not mention pain [1].

## Resources

### Framing questions

- What is the cost and/or resource requirements of the intervention? (INTEGRATE + adapted to include resource requirements)
- What is the overall budget impact of implementing the intervention? (INTEGRATE)
- Do cost and budget impacts vary in the short- versus longer-term, and are they sustainable? (INTEGRATE)
- What is the value-for-money of the intervention, based on an appropriate choice of method, e.g. cost-effectiveness, cost-benefit or cost-utility? (INTEGRATE)

### Induction of labour at or beyond 37 weeks' gestation

##### Study characteristics

Economic evidence is very limited, derived from two trial-based primary studies conducted in high-income settings. Any conclusions drawn from these studies should be viewed as extremely tentative.

Two **trial-based economic studies** evaluating costs or healthcare resource utilisation were reviewed. Detailed characteristics and results are included in Additional file 2.

Goeree 1995 used a cost-minimization analysis to calculate cost differences in the period from inpatient admission to maternal or neonatal discharge (intrapartum period) from a Canadian healthcare system perspective [4]. Costs were expressed in 1992 Canadian dollars. Effectiveness and resource utilization data were derived from Hannah 1992, an RCT conducted in 22 Canadian hospitals comparing induction of labour with serial fetal monitoring while awaiting spontaneous labour in women with uncomplicated pregnancies of 41 or more weeks’ gestation [5].

Grobman 2020 assessed healthcare resource utilization from a US hospital perspective across three periods: antepartum; delivery admission, or intrapartum period; and from discharge to 8 weeks postpartum [6]. Resource utilization data were derived from Grobman 2018 (the ARRIVE study), an RCT conducted in 41 US hospitals comparing induction of labour at 39 weeks’ gestation with expectant management in low-risk nulliparous women [7].

Both Hannah 1992 and Grobman 2018 are included in the 2020 Cochrane review of induction of labour at or beyond 37 weeks' gestation [8].

Three eligible **model-based economic studies** were also identified by Eddy et al [9].

Kaufman 2002 used a decision-tree model of a hypothetical cohort to compare costs of labour induction at 39 weeks’ gestation versus expectant from a health sector perspective, using 2011 cost data from published literature [10].

Kaimal 2011 compared costs/QALY of labour induction at 41 weeks’ gestation versus expectant management using US National Birth Cohort data and 2007 cost data from published literature. The perspective was not specified [11].

Hersh 2019 compared costs and QALYs of labour induction at 39 weeks’ gestation versus expectant management using US 2016 National Vital Statistics and 2018 from local sources and published literature [12].

##### Cost

In Goeree 1992, the mean cost per woman in the induction group CAD 2939 (95% CI: 2898 to 2981) compared with CAD 3132 (95% CI: 3090 to 3174) per woman in the spontaneous labour group. The mean cost saving per woman with labour induction was CAD 193 (95% CI: 133 to 252) [4].

##### Resource usage

Grobman et al reported that women who underwent labour induction used fewer resources in the antepartum period (routine prenatal care, unscheduled outpatient visits, inpatient admissions, fetal assessments, laboratory testing and other treatments, such as IV hydration and antibiotics).

In the intrapartum period, resource usage between the two groups was more balanced overall, with differences in the types of resources used. Women in the labour induction group were more likely to have a longer duration in delivery and labour, but shorter inpatient stays after delivery. They had more use of cervical ripening agents, oxytocin infusions and intrauterine pressure catheters, but less use of magnesium sulfate and parenteral antibiotics. Neonates born to women in the labour induction group had shorter inpatient admissions and were less likely to use CPAP or high-flow oxygen.

In the postpartum period, resource usage between the two groups was largely similar. Neonates born to women in the labour induction group were more likely to use outpatient serum bilirubin testing, however the authors thought this was likely due to their shorter inpatient stays [6].

##### Overall budget impact

From a health system perspective, Goeree et al estimated that the savings in Canada from implementing a universal policy of post-term pregnancy management by labour induction could be as high as CAD 8 million per year. This estimate was based on lower monitoring costs and rates of caesarean among women in the labour induction group. The study suggests these savings would likely be higher if a wider cost perspective (e.g. patient expenses and quality of life) was used [4].

##### Value-for-money

The trial-based economic studies did not undertake value-for-money analyses. Goeree et al undertook a cost-minimization analysis, as there was no difference in perinatal mortality and neonatal morbidity in the Hannah 1992 RCT [4]. Grobman et al did not undertake a cost analysis, however they concluded that the better maternal and neonatal health outcomes associated with labour induction for post-term pregnancy can be gained without greater healthcare resource utilization across the antepartum, intrapartum and postpartum periods [6].

### Outpatient induction of labour

##### Study characteristics

Economic evidence is very limited, derived from one **trial-based primary study** conducted in a high-income setting. Any conclusions drawn from this study should be viewed as extremely tentative. Detailed characteristics and results are included in Additional file 2.

Adelson 2013 undertook a cost analysis from an Australian hospital perspective in the period from priming until maternal or neonatal discharge. Overall cost data were extrapolated from the actual costs at one participating hospital and expressed in 2010-11 Australian dollars [13]. Effectiveness data were derived from Wilkinson 2015 (the OPRA study), an RCT conducted over three years (2008-2011) in two Australian hospitals comparing outpatient versus inpatient of labour using vaginal PGE2 gel in 823 low-risk women with prolonged pregnancy. The median gestational age at priming was 40 weeks and 6 days [14].

Wilkinson 2015 is included in the 2020 Cochrane review of home versus inpatient induction of labour for improving birth outcomes [15].

One eligible **model-based economic study** was also identified by Eddy et al [9].

Son 2020 used a decision tree model to determine cost savings, cost per patient and incremental cost of outpatient versus inpatient ripening with transcervical balloons in term (≥ 37 weeks) singleton pregnancies with unfavourable cervix, using costs from published literature and a US healthcare system [16].

##### Cost

Adelson et al found the cost saving for women randomised to the outpatient group was AUD 319 per woman (95% CI: 104 to 742). When restricting the analysis to women who actually received the intervention (i.e. excluding those with spontaneous onset of labour), the in-hospital costing saving was AUD 433 (95% CI: 282 to 1148), however the cost of the outpatient priming clinic reduced the overall cost saving to AUD 156 per woman. The authors noted that only approximately half of all the women in the outpatient priming group were discharged home or remained home overnight once they received the PEG2 gel. They suggested using cervical priming techniques that do not cause contractions may lead to more women returning and/or remaining at home with greater cost savings than shown in this study [13].

##### Overall budget impact

Overall budget impacts of a policy of outpatient labour induction in low-risk at or beyond term women were not analysed in Adelson et al [13].

##### Value-for-money

The Adelson 2013 trial-based study undertook a cost analysis only. A cost-utility or cost-effectiveness approach was not considered suitable by the authors due to the short time horizon and lack of QoL measure in the OPRA study [13].

### Mechanical methods for induction of labour

##### Study characteristics

Economic evidence is very limited, derived from two **trial-based primary studies** conducted in high-income settings. Any conclusions drawn from this study should be viewed as extremely tentative. Detailed characteristics and results are included in Additional file 2.

ten Eikelder 2018 undertook a cost-effectiveness analysis from a hospital perspective in the period from admission to antenatal care ward to discharge. A composite safety outcome (asphyxia and postpartum haemorrhage) and caesarean section were used as effectiveness outcomes. Cost data were estimated using different methods and sources and expressed in 2013 Euros [17]. Effectiveness data were derived from ten Eikelder 2016 (the PROBAAT-2 study), an RCT conducted in six tertiary and 23 secondary care hospitals in the Netherlands comparing oral misoprostol (50 mcg every 4 hours, with a maximum three doses in 24 hours) with Foley catheter for labour induction in 1845 women at term with an unfavourable cervix and without previous caesarean section [18].

van Baaren 2013 undertook a cost-effectiveness analysis from a hospital perspective in the period from admission to antenatal care ward to six weeks postpartum. A composite safety outcome (asphyxia and postpartum haemorrhage), caesarean section and neonatal admission were used as effectiveness outcomes. Cost data were estimated using different methods and sources and expressed in 2009 Euros [19]. Effectiveness data were derived from Jozwiak 2011 (the PROBAAT study), an RCT conducted in twelve hospitals in the Netherlands comparing Foley catheter with vaginal prostaglandin E2 gel for labour induction in 819 women at term with an unfavourable cervix and without previous caesarean section [20].

Both ten Eikelder 2018 and van Baaren 2013 are included in the 2019 Cochrane review of mechanical methods for induction of labour [21].

One eligible **model-based economic study** was identified by Eddy et al [9].

Alfirevic 2016 undertook a cost-effectiveness analysis using a decision-tree model comparing vaginal PGE2 tablets (reference intervention) with 19 other mechanical and drug labour induction methods from a UK healthcare system perspective. Cost data were derived from NHS reference data and other sources, and expressed in 2012-13 British pounds. Utility estimates were derived from the literature. Effectiveness data were derived from the authors’ network meta-analysis of RCTs of women eligible for third-trimester labour induction [22].

##### Cost

ten Eikelder et al reported a mean cost per woman of 4158 (2013 Euros) when using Foley catheter for labour induction, which was comparable to oral misoprostol costs. Costs were comparable with varying resource use and unit price estimates. Sensitivity analyses showed that overall costs were lower for nulliparous women induced with Foley catheter (4561 versus 5126), but higher for multiparous women (3419 versus 3136). This was mainly due to the shorter time from induction to delivery in multiparous women [17].

van Baaren et al found that mean costs per woman of 3297 (2009 Euros) in the Foley catheter group were not significantly higher than for women induced with PGE2 gel. While the induction materials were less expensive in the Foley catheter group, the average duration of stay on the labour ward was longer than for women induced with PGE2 gel. A sensitivity analysis showed that PGE2 induction in multiparous women resulted in lower costs, mainly due to the shorter time from induction to delivery than for nulliparous women [19].

##### Overall budget impact

Overall budget impacts of a policy of mechanical methods for labour induction were not analysed in the trial-based studies.

##### Value-for-money

ten Eikelder et al did not find the incremental cost-effectiveness ratios (ICER) for the composite outcome of asphyxia and post-partum haemorrhage to be informative due to comparable efficacy between the interventions. The ICER to prevent one caesarean section using oral misoprostol was 10,338 (2013 Euros), however this was not found to be significant. At a willingness-to-pay threshold of at least 30,000 per woman, oral misoprostol may be cost-effective for caesarean section [17].

van Baaren et al did not find the ICER for caesarean section to be informative due to comparable efficacy between the interventions. Using a Foley catheter, the ICER to prevent one neonatal admission was 2708 (2009 Euros), and 5257 to prevent one composite outcome of asphyxia and post-partum haemorrhage. It is increasingly probable that labour induction with Foley catheter is cost-effective when increasing the willingness-to-pay threshold [19].

## Equity

### Framing questions

- Is the intervention likely to reduce or increase existing health inequalities and/or health inequities? (INTEGRATE)
- Does the intervention prioritise and/or aid those furthest behind? (INTEGRATE)
- How do such impacts on health inequalities and /or health inequities vary over time, e.g. are initial increases likely to balance out over time, as interventions are scaled up? (INTEGRATE)
- How are the benefits and harms of the intervention distributed across the population? Who carries the burden (e.g. all), who benefits (e.g. a very small sub-group)? (INTEGRATE)
- How affordable is the intervention for individuals, households or communities? (INTEGRATE)
- How will it impact household health expenditures, including risk of catastrophic health expenditures and health-related financial risks? (INTEGRATE)

### Research evidence

No direct evidence was identified to address these questions. The 2015 WHO report on inequality in reproductive, maternal, newborn and child health documented significant inequalities in low- and middle-income countries [23]. While there has been improvement, particularly in reducing within-country gaps for disadvantaged groups, the report states that “the poorest, the least educated and those residing in rural areas have lower health intervention coverage and worse health outcomes than the more advantaged” [23, p. xii]. These groups are also likely to have the highest maternal healthcare needs. The report states that preventing and reducing morbidity and mortality in childbirth can play a key role in reducing overall health inequities, given the critical juncture childbirth represents in lifelong health. The 2018 WHO update of labour induction at or beyond term considered “[s]afe, effective, equitable implementation of this intervention to prevent perinatal mortality and morbidity could therefore reduce health inequities” [24, p. 31].

##### Socio-economic status and education

A secondary analysis of WHO Global Survey on Maternal and Neonatal Health provides support for the role of educational status in health inequities. This analysis showed that women in both Asia and Africa regions undergoing labour induction were more likely to have ten or more years of education [25].

The studies included in the Coates 2019 QES were all conducted in high-income settings. Educated patients of higher socio-economic status tend to participate more frequently in their healthcare, access more healthcare services during their perinatal period, and as a result have measurably better health outcomes [26, 27]. It is therefore likely that women from low- and middle-income settings, or disadvantaged groups within a high-income setting, will experience greater barriers to participation in healthcare decision-making than indicated in the QES findings [28].

The QES findings indicate that women have a range of views regarding the acceptability of induction of labour in terms of the intervention itself, the timing of induction, and the induction setting (outpatient vs inpatient) (see **Acceptability** below).

Issues of inequity may arise if women’s choices about induction are not fully supported. Barriers to active participation in decision-making include status and power differentials between healthcare professionals and women making decisions about undergoing labour induction; differing beliefs, expectations, knowledge, values and preferences; and having the confidence and/or ability to challenge or confirm medical opinions and navigate the health system [27].These barriers can be more complex for women from non-dominant cultural backgrounds and/or where the dominant language spoken is not their first language

In order to ensure that implementation of labour induction does not reinforce existing inequities, it is important that all women have access to full information presented in lay terminology and in a timely manner; are resourced to use their own social networks to assist them understand the information presented if required; and, as much as is feasible, are able to have continuity of care to ensure a woman’s healthcare provider is aware of her needs, values and preferences [27].

##### Geography and country income-setting

WHO general principles of induction of labour outline that the intervention should only be carried out where facilities for assessing maternal and fetal-wellbeing and performing caesarean sections are available. Additionally, women receiving oxytocin, misoprostol and other prostaglandins should never be left unattended [24].

Women living in low-middle income settings and/or remote or rural areas may be less likely to have access to these facilities. The facilities in their settings may also be under-resourced and unable to provide the level of monitoring and assessment required [29]. This may reduce their ability to access labour induction for safe birth and delivery, leading to poorer health outcomes, and reinforcing existing health inequality.

In an additional study of clinician views of labour induction, scheduling inductions to ensure minimal disruption to their general practice was one view given by rural GPs in Australia. Rural midwives also reported that resource and facility limitations and the associated patient safety concerns impacted on labour induction availability in their setting [30].

##### Antenatal care visits

In 2016, WHO recommended that women receive one ultrasound scan before 24 weeks gestation in order to provide more accurate estimation of gestational age when considering labour induction [31]. Findings from both Vogel 2013 and a 2015 WHO report show that inequality in antenatal care (ANC) coverage (of either one or the WHO recommended four ANC visits) remains for all three dimensions of inequality (economic status, education and place of residence) [23, 25].

The recently updated Cochrane reviews did not identify evidence which would enable assessment of whether the balance of benefits and harms of labour induction varies in different population subgroups [8, 32, 33].

##### Affordability

Universal healthcare (UHC) is essential so people can access necessary interventions such as labour induction without experiencing financial hardship. Most essential coverage gaps exist in low-income countries, particularly on the African continent. However, based on their larger populations, lower-middle-income countries have the most people lacking coverage. Financial hardship due to out-of-pocket health spending usually results from outpatient medicines and care, rather than hospital bills. Many countries offer fee exemptions at public facilities for maternal health services, such as normal deliveries, caesarean sections and management of other birth complications [28, 34].

##### Impacts of scale-up

Rates of labour induction in high-income settings are increasing, and some low-middle income settings are also seeing a rise in labour induction rates comparable to high-income settings [29]. As labour induction becomes more widely available and more women access this intervention, the benefits of labour induction for improved maternal and neonatal health outcomes may help reduce existing health inequalities and/or health inequities.

## Acceptability & human rights

### Framing questions: acceptability

- Is the intervention acceptable to key stakeholders? (INTEGRATE)
- Is the intervention sensitive to sex, age, ethnicity, culture or language, sexual orientation or gender identity, disability status, education, socio-economic status, place of residence or any other relevant characteristics?

### Research evidence: acceptability

##### Acceptability to women

Findings from the QES and additional primary studies indicate that:

- women hold widely varying (and sometimes contradictory) views on the acceptability of induction of labour
- induction of labour is more acceptable to women when there is a recognised need to avert harm to the baby
- acceptability varies according to women’s trust in their healthcare provider, their perception of birth as a natural process, their need for certainty, and the duration of waiting (i.e. extent of post-term pregnancy).

In the Coates 2019 QES, some women expressed trust and confidence in their healthcare provider to “know best” (direct quote from participant extracted from Coates 2019) and were therefore accepting of induction of labour when recommended to them. Other women were happy to undergo induction of labour in order to have some certainty of their pregnancy ending and/or relieve feelings of discomfort. Some women felt concerned that induction of labour would lead to further interventions and were unhappy they would not experience spontaneous onset of labour. Women who felt scared or hesitant about induction of labour, were still willing to undergo the intervention if it reduced the risk of harm to their baby [1].

An additional study interviewed women during their third trimester and again in the postpartum period. In the pre-birth period, women who were planning to birth with a lay birth attendant or unassisted at home were either opposed to induction of labour altogether, or would only agree to it if there were risks to mother and/or baby if the pregnancy continued. In their view, it was best to let nature take its course [35].

Almost all of the women in Westfall 2004 who experienced pregnancies that continued beyond 40 weeks chose proactive measures to stimulate labour, but only after they had passed 40 weeks gestation. These women did not consider prolonged pregnancy to be a medical issue, and preferred to ‘take matters into their own hands’, rather than undergo a medical induction. This included attempting to induce labour with castor oil, essential oil suppositories, homeopathic preparations or by having intercourse [35].

An additional study (Wessberg 2017) captured women’s experiences of pregnancy at or beyond 41 weeks gestation. Many contradictory and conflicting feelings were reported, both between women and within each woman. While women longed for the arrival of their baby, they also did not want labour to begin too quickly if the baby wasn’t ready. These feelings often changed as the pregnancy progressed, moving from a feeling of trust in their body and confidence their baby would arrive when it was ready, to feeling insecure in their ability to give birth and frustrated that labour would never begin. Over time, their concern for their baby increased as they wondered if there was something wrong with their baby, or their body, that was delaying labour onset. Women acknowledged that these contradictory feelings were to be expected, however they experienced their lack of control over when the birth would happen as difficult [2].

We did not find any direct evidence of differences in socio-cultural acceptability of labour induction in relation to any of these characteristics in the Coates 2019 QES or additional studies.

##### Acceptability to implementers

There is limited evidence available on the acceptability of induction of labour to clinicians and more research would be useful to inform recommendations.

A study of obstetrician and midwife opinions on induction of labour found that obstetricians perceived a lack of clear evidence on the risks and benefits of labour induction to guide their decision-making. They were particularly concerned about neonatal safety and the potential for medical litigation, and were uncertain about the risks of caesarean birth following induction. They also expressed uncertainty and reported practice variation in relation to the optimal timing of induction [30].

The available evidence does not address whether the acceptability of the labour induction varies with socio-cultural factors.

##### Acceptability to women of outpatient labour induction

In the Coates 2019 QES, some women undergoing outpatient labour induction reported valuing the access to social support being at home enabled. Some women also valued a sense of control that was partly due to the freedom to move around as they needed. It was important for them to be able to carry on with their daily activities, or activities of their own choice, while waiting for the onset of labour. These women reported that being comfortable at home, including “eating, sleeping, moving and bathing in familiar ways” [1, p 25], helped distract them from being ‘in limbo’ while waiting for labour to start. Outpatient induction also meant women could continue with their usual caregiving and spend time with older children before their birth. Women said that being able to continue with these aspects of daily life also helped them cope with their contractions.

Some women who underwent outpatient IOL had additional safety concerns about going home, particularly that the onset of labour might be very sudden, and whether they would be able to recognise if something was wrong.

The QES authors found that “outpatient labour induction is not preferable for all women, and individuals will have preferences about what constitutes a comfortable and safe environment for labour” [1, p 26]

### Framing questions: human rights

- Is the intervention in accordance with universal human rights standards and principles? (INTEGRATE)

### Research evidence: human rights

In 2012, the UNCHR provided guidance on applying a human rights-based approach when developing and implementing policies and programmes designed to reduce maternal mortality and morbidity. These health rights include claims for health goods and services, and extend to the right to information that enables women to be active agents when making decisions that affect their health [36].

The WHO Quality of Care Framework for pregnant women and newborns highlights three key ‘experience of care’ processes that uphold and protect health and human rights: effective communication, respect and preservation of dignity, and emotional support [37].

The findings from the Coates 2019 QES and additional studies in populations and stakeholder groups relevant to induction of labour highlight a gap between the rights of women to their reproductive health and the experiences of women who are considering, or have undergone, induction of labour. Overall, women wanted more timely and complete information about the risks and benefits, or process, of induction of labour to enable them to make a competent, informed and voluntary decision about the intervention. In these studies, there was a perception that the timing and decision to induce labour was determined by facility or system constraints, rather than being patient-centred. This left women feeling overlooked, ignored and without support. These findings highlight the vulnerable state women can experience in their immediate pre-birth period, and the need for a healthcare system which enables clinicians to provide appropriate reassurance, listening and complete and high-quality information.

More detailed findings on these key aspects are given below, and are in broad agreement with the main findings of a 2018 QES of what matters to women during childbirth:

*“What mattered to most women was a positive experience that fulfilled or exceeded their prior personal and socio-cultural beliefs and expectations. This included giving birth to a healthy baby in a clinically and psychologically safe environment with practical and emotional support from birth companions, and competent, reassuring, kind clinical staff. Most wanted a physiological labour and birth, while acknowledging that birth can be unpredictable and frightening, and that they may need to `go with the flow'. If intervention was needed or wanted, women wanted to retain a sense of personal achievement and control through active decision-making.” [38, p. 1]*

##### Information provision and effective communication

In most of the studies included in the QES, many women reported wanting more information from healthcare providers about the reason for induction, the process of induction and the risks and benefits of induction specific to their circumstances. Some women said that they wanted more timely, balanced and full information on the risks and benefits, which would enable them to make an informed choice and avoid feelings of resignation about their lack of role in decision-making. Some women described being told their ‘time had run out’ and that they would be induced.

Many women also reported a desire for more information about the process of labour induction to help them prepare for the intervention, including: whether and how long they would be in hospital before their onset of labour; how medication would be administered; what delays they might experience; how long the process might last; if they would be on a ward or in a birthing centre; how severe the pain might be; and if their support person/people could stay with them overnight.

Women value provision of information early to enable them to process the information and consider their options. Women suggested that provision of information in antenatal classes would be beneficial, and noted that provision of information during the labour induction booking process did not provide time to consider the information or be involved in the decision-making.

Women reported turning to the internet, books or friends and family to seek information to inform their decisions about labour induction and noted that non-health factors, such as a partner’s vacation time, also informed their decision-making.

Women valued provision of information about labour induction in the context of a broader conversation about post-term pregnancy, rather than a single decision about induction of labour. They also valued the opportunity to discuss the information with a healthcare provider and to have their thoughts and feelings heard.

The additional study of obstetrician and midwife views of labour induction found that obstetricians tended to focus more on providing information about the process of labour induction, rather than its risks and benefits. Midwives reported that time constraints in the clinic reduced their ability to provide information about labour induction during antenatal visits. They indicated that antenatal education needed to improve to ensure women fully understood the risks and benefits of labour induction, as well as the process itself. In general, clinicians expressed uncertainty that women were fully informed and therefore had the capacity for autonomous decision-making in relation to labour induction [30].

##### Privacy, dignity and control

Women valued setting and systems that provided them with privacy, dignity and control.

In the Coates 2019 QES, some women in inpatient settings felt disturbed by the lack of privacy and its impact on their ability to rest, sleep and focus on their induction experience. They found the ward setting to be noisy and busy, with the sounds of other women, presence of other women’s support people (‘strangers’), bright lights and other technology. They were also worried about the effect their labouring noises might have on other women. Women also noted that even if they had support from family and friends, the lack of privacy meant they were unable to fully appreciate that support.

Women wanted a system that would ensure they were consistently cared for, continually monitored, could communicate their needs and have them responded to. Some women said that even though they were surrounded by others, they felt forgotten, isolated and alone. They were aware that midwives were busy, and felt unable to bother them. Some were angry that when they did report pain or that their labour had progressed, they were not believed or listened to. They felt like they were “lined up, part of a checklist” [1, p 24], rather than an individual. Other women experienced loss of control when they were moved to other parts of the hospital without prior knowledge.

In the Coates 2019 QES, women reported that undergoing induction in an inpatient setting wanted greater freedom of movement. For some women, needing to be hospitalised to undergo induction evoked feelings of dread, fear or panic. For others, being induced in hospital was experienced as being comfortable, as it meant being relieved of having to take care of anyone else.

##### Emotional support

Women valued a sense of security. It was important for women’s sense of security to have a support person present during labour induction. When this was not possible in an inpatient setting, it evoked feelings of anxiety, fear and isolation. Some women experienced undergoing labour induction in hospital as a place of safety and security, knowing they had immediate access to healthcare providers and technology.

## Feasibility

### Framing questions: infrastructure

- How does the intervention interact with the need for and usage of the existing health system infrastructure (e.g. types of health facilities, health information system, medical products and technologies) at national and subnational levels? (INTEGRATE)
- Is it likely to impact on these and their performance in positive or negative ways? (INTEGRATE)

### Research evidence: infrastructure

No direct evidence was identified to address these questions.

A 2020 cross-sectional analysis of childbirth interventions in 13 high-income OECD countries reported 2013 labour induction rates ranging from 13.8% to 35.9% of all births [39].

Secondary analyses of WHO Global Survey on Maternal and Neonatal Health data found labour induction 2004-2005 rates in Latin America ranging from 5.1% to 20.1% [40] and in African countries from 1.4% to 6.8% [25]. While rates on the African continent are relatively low, it is estimated that the unmet need for medically indicated inductions ranges from 66% to 80% [29]. The 2007-08 labour induction rates in Asian countries ranged from 2.5% to 35.5%. Elective inductions in most African and Asian countries accounted for less than 2% of all inductions (the most significant exceptions being in Sri Lanka and Japan, where elective inductions accounted for 27.8% and 8.5% of all inductions respectively) [25]. Whereas in Latin America, elective induction rates were 29% of all inductions [40].

These rates indicate labour induction is widely implemented for medical indications and elective reasons.

Oxytocin alone remains the most frequent labour induction method in all income settings. Use of prostaglandins and particularly misoprostol in lower- and middle-income countries is not widespread, accounting for approximately 15% of inductions in the African and Asian regions [25]. In Latin American countries, misoprostol was used in approximately 9% of all elective labour induction deliveries, and other prostaglandins were rarely used [41]. The higher cost of prostaglandins, other than misoprostol, may limit their affordability and use in low-income settings [29].

Misoprostol is on the WHO Essential Medicine List for labour induction [42], however in many countries it is not licenced for obstetric use. Off-label use of misoprostol for labour induction is possible in some countries [29], while in other countries, misoprostol may not be available at all [43]. While misoprostol is low-cost, heat stable and easy to administer and store , in most countries it is only available in a 200 mcg tablet. WHO recommends an initial dose of 25 mcg for labour induction, and this may lead to safety issues in settings where low-dose tablets are not available, and solutions for low-dose administration are made from higher dose tablets [29].

Electronic fetal heart rate monitoring equipment is required, as is access to a health facility where caesarean sections can be performed.

Performing induction of labour safely requires availability of appropriate drugs or mechanical devices, monitoring equipment and access to facilities for safe caesarean section. Inconsistent supply, or lack of, drugs and medical equipment and access to appropriate facilities may be an issue in some lower-income settings and/or rural settings.

### Framing questions: health workforce

- How does the intervention interact with the need for and usage of the existing health workforce? (INTEGRATE)
- Is it likely to impact on these in positive or negative ways, for example by affecting the number or distribution of staff, their skills, responsiveness or productivity? (INTEGRATE)

### Research evidence: health workforce

No direct evidence was identified to address these questions.

##### Antenatal care

WHO recommends that women receive one ultrasound scan before 24 weeks gestation in order to provide more accurate estimation of gestational age when considering labour induction [31]. This requires trained healthcare workers in the antenatal care setting.

Findings from the Coates 2019 QES also indicate that women would like to receive information about the risks and benefits, as well as process, of labour induction during their ANC visits [1]. Midwives in an additional primary study of clinician views of labour induction reported that time constraints in the ANC clinics were a barrier to providing this information [30].

Trained healthcare worker shortages in lower- and middle-income country settings are also a significant barrier to the provision of ANC for risk assessment and information provision [23].

##### Performing labour induction

WHO general principles for performing labour induction state:

- Wherever induction of labour is carried out, facilities should be available for assessing maternal and fetal well-being
- Women receiving oxytocin, misoprostol or other prostaglandins should never be left unattended
- Wherever possible, induction of labour should be carried out in facilities where caesarean sections can be performed [24]

Findings from the Coates 2019 QES of studies undertaken in high-income settings indicate that women in inpatient settings can feel alone and forgotten once induction has begun. They were aware that midwives were busy and at times unable to attend to them [1].

Given the acknowledged shortage of skilled birth attendants in many low- and middle-income country settings [23], healthcare workers in these settings may be required to attend to much higher numbers of women on the labour ward than in other settings . Under-resourced settings may face significant challenges in providing the required level of assessment and monitoring during labour induction [29].

There are also additional requirements for surgical obstetric and operating theatre staff for women who require caesarean section delivery if labour induction is not successful. Staff to transfer women to facilities with these capabilities may also be required, particularly in rural areas.

Ensuring women are provided with the information needed to make an informed decision about labour induction during ANC visits requires additional staff time. This may impact negatively on the number of ANC visits that can be provided in a facility.

Staff requirements for monitoring and assessment, and provision of supportive care for women undergoing labour induction may affect the number of skilled birth attendants required and/or their responsiveness, particularly in under-resourced settings.

A higher number of induction deliveries are attended by medical doctors than non-induction deliveries [25]. This has implications for the distribution and productivity of medical doctors, particularly in under-resourced settings.

##### Additional considerations for outpatient labour induction

For women undergoing outpatient labour induction, availability of staff to answer questions and reassure women [1], as well as assess when women should return to the inpatient setting, is required. For women experiencing adverse reactions, such as uterine hyperstimulation, in the outpatient setting, attendance by paramedics for care and transfer to a healthcare facility may be required [32].

# Search strategies

## PubMed

(Labor, Induced[Mesh Terms] OR "induction of labour" OR "induction of labor" OR "induced labour" OR "induced labor" OR "labour induction" OR "labor induction" OR "labour induced" OR "labor induced") AND (Systematic[SB] OR meta-analysis[Publication Type] OR Cochrane Database Syst Rev[SO] OR review[Title] OR "systematic review"[Text Word] OR "rapid review"[Text Word] OR "scoping review"[Text Word] OR "living review"[Text Word] OR "meta-analysis"[Text Word] OR "qualitative evidence synthesis"[Text Word]) AND 2018:3000[DP]

## Epistemonikos

"induction of labour" OR "induction of labor" OR "induced labour" OR "induced labor" OR "labour induction" OR "labor induction" OR "labour induced" OR "labor induced" in (Title/abstract) with filter for 2018:2022 and Systematic reviews.

# Qualitative evidence eligibility and quality assessment

## QES eligibility assessment

The criteria below were used to assess QES eligibility during title and abstract, and full-text review in Covidence. Criteria three and four are aligned with domains three and four of the ENTREQ reporting guidelines.

1. Primary focus on induction of labour
2. Titled or expressly stated to be a systematic review
3. Documented search and selection criteria

- Was the search approach documented (e.g. pre-planned or iterative until saturation achieved)?
- Were the inclusion criteria were specified and justified (e.g. population, language, year limits, study type?
- Were data sources and search strategy described?
- Was the process of study selection described?
- Were study characteristics provided (including reasons for exclusion)?

1. Appraisal of included studies for reliability using a documented method

- Was the rationale for the appraisal was documented (e.g. assessment of conduct, reporting and/or utility of findings)?
- Were tools, frameworks and criteria for appraisal were documented (e.g. existing tools, reviewer developed tools, description of domains assessed)?
- Was the process of quality appraisal described?
- Were the appraisal results presented (including rationale for any weighting/exclusion based on the quality assessment)?

1. Findings relevant to any EtD criteria

Figure 1 below shows the PRISMA flow diagram the first stage of eligibility screening for QES.

**
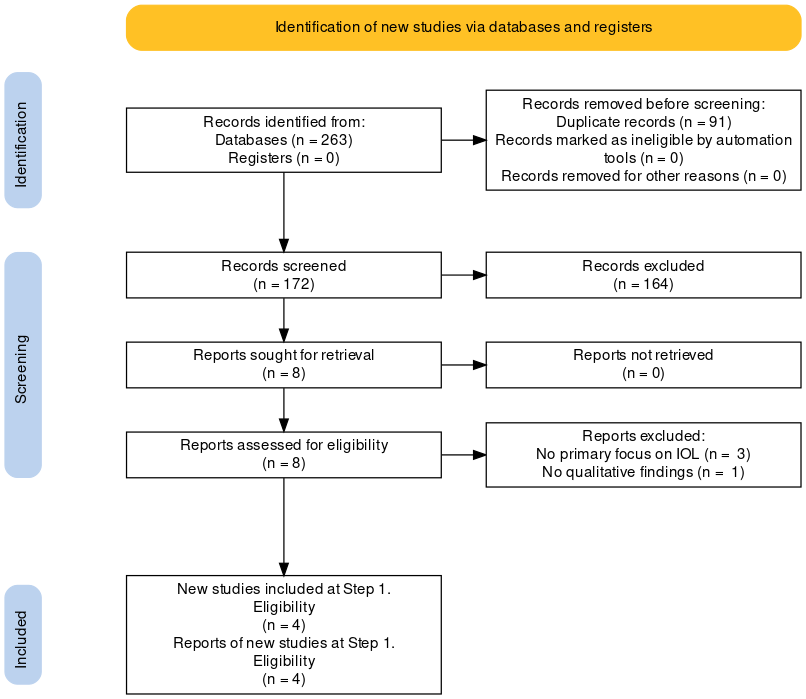
**Figure 1: PRISMA flow diagram – QES eligibility screening

## QES quality assessment

Eligible QES were further assessed for quality using the following domains informed by the ENTREQ statement. We also documented if an approach was used to assess certainty of findings, e.g. GRADE CERQual [44].

1. Introduction, methods, methodology

- Was the research question was clearly stated?
- Was the synthesis methodology and rationale for its selection documented?
- If quotations from primary studies were provided, was it clear whether they were direct quotations from participants, or author interpretation?

1. Synthesis of findings

- Were the sections of the included studies that were analysed defined, and was data extraction described?
- Was the process of coding, comparison and derivation of themes described?

### Domain judgements

Questions in each domain were assessed as Yes, No or Partial and an overall judgement made for each domain as outlined below.

- High: all criteria in domain = Yes
- Moderate: most criteria in domain = Yes/Partial
- Low: most criteria in domain = Partial/No
- Very low: most criteria in domain = No

# Qualitative evidence selection

A 2019 QES of women’s experiences of induction of labour (Coates 2019) [1] was included in the EtD on the basis of quality (high) and greatest breadth of qualitative primary studies compared with two other eligible QES [45, 46] and one scoping review [47]. Primary studies not included in Coates 2019 were screened for additional relevant findings and/or income-settings and/or clinician views. Five additional studies were identified and relevant findings extracted for inclusion in the EtD [2, 3, 30, 35, 48].

Characteristics of included qualitative studies can be found in [section 7](#_Characteristics_of_included). Characteristics of participants in the included qualitative studies can be found in [section 8](#_Participant_characteristics_in).

# Cost and cost-effectiveness evidence

Eligible primary studies were identified from a WHO living scoping review of cost-effectiveness evidence for maternal and perinatal health interventions [9]. Review, data extraction of detailed findings and quality appraisal was undertaken for trial-based economic studies only. Brief characteristics of model-based economic studies have been noted. Results from model-based studies have not been included in the findings due to the numerous assumptions underlying these and the difficulty of determining their generalizability. More detailed study characteristics and data extraction for trial-based economic studies are provided in Additional file 2.

# Preparation of EtD sections

The process of preparing the EtD sections was undertaken in a series of stages: data extraction, thematic coding and analysis.

One researcher extracted all relevant findings from the selected QES, additional primary qualitative studies, economic studies and additional evidence and framing information for the criteria of equity, socio-cultural acceptability (human rights) and feasibility into separate tables for each of the WHO-INTEGRATE EtD criteria, and mapped to the sub-criteria and framing questions. Some findings were relevant to more than one criteria, sub-criteria or framing question. Any questions about the inclusion of findings in the initial tables were discussed and resolved with a second researcher.

The findings were then organised into themes for each question as suggested by the data by a single researcher, then discussed with two other researchers and revised as necessary. A single researcher then drafted the detailed findings sections for each EtD criteria/sub-criteria/question, identifying which findings were common to all three topics, and which were specific to a single topic. The draft detailed findings were reviewed by the second researcher, who provided a second draft for discussion and agreement. This draft was reviewed by a third researcher to check inclusion and interpretation of findings from the original tables.

Once agreement on the detailed EtD section text was reached by all researchers, a summary of the detailed text for each EtD section was prepared by a single researcher and agreed to by a second researcher through iterative discussion. The WHO lead then reviewed the detailed and summary findings, providing feedback which was incorporated into the final EtD section text of the EtD documents provided to the GDG.

# EtD Framework mapping

Note: headings of sections which were not applicable have been indicated with type *in italics*

| **WHO-INTEGRATE** | **GRADE** | **Comments** |
| --- | --- | --- |
| 1. Balance of health benefits and harms | **2. Values** |  |
| **Patients’/beneficiaries’ values in relation to health outcomes** |  |  |
| To what extent do patients/beneficiaries value different health outcomes? | Is there important uncertainty about or variability in how much people value the main [health] outcomes?  Note: In 2018 WHO IOL update, this question was included in the EtD (Annex 4) under 1. Effect of interventions. There was no separate section for Values. | Also considered these questions from the WHO Handbook for Guideline Development [49]:  How important are the health outcomes linked with an intervention to the people affected?  Which outcomes matter most?  How do they feel about the possible benefits vs the possible harms of labour induction? |
| **2. Human rights & socio-cultural acceptability** | **5. Acceptability** |  |
| **Accordance with university human rights standards and principles** |  |  |
| Is the intervention in accordance with universal human rights standards and principles? | The GRADE paper (EtDs for CPGs) does refer to principles (e.g. autonomy, justice) that indicate inclusion of human rights perspective can be considered here [50]. | Human rights question presented second in findings for this criteria |
| **Socio-cultural acceptability of intervention to patients/ beneficiaries and those implementing the intervention** |  |  |
| Is the intervention socio-culturally acceptable to patients/beneficiaries as well as to those implementing it? | Is the intervention acceptable to key stakeholders? | Acceptability question presented first in findings. |
| To which extent do patients/beneficiaries value different non-health outcomes? |  |  |
| **Socio-cultural acceptability of intervention to the public and other relevant stakeholder groups** |  |  |
| Is the intervention sensitive to sex, age, ethnicity, culture or language, sexual orientation or gender identity, disability status, education, socio-economic status, place of residence or any other relevant characteristics? |  | No evidence found. |
| ***Impact on autonomy of concerned stakeholders*** |  |  |
| *How does the intervention affect an individual’s, population group’s or organization’s autonomy, i.e. their ability to make a competent, informed and voluntary decision?* |  | This sub-criteria relates to autonomy in terms of: to what degree is the intervention imposed on individuals/communities/populations (e.g. public health orders during a pandemic)? This sub-criteria was not considered relevant to the intervention of induction of labour. |
| **3. Health equity, equality and non-discrimination** | **4. Equity** |  |
| **Impact on health equality and/or health equity** |  |  |
| *How are the condition and its determinants distributed across different population groups?* | What would be the impact on health equities? | In our context, the condition might be ‘need for labour induction’. There is no scope to consider this question for our topics. |
| Is the intervention likely to reduce or increase existing health inequalities and/or health inequities?  Does the intervention prioritise and/or aid those furthest behind? |  |  |
| How do such impacts on health inequalities and /or health inequities vary over time, e.g. are initial increases likely to balance out over time, as interventions are scaled up? |  |  |
| **Distribution of benefits and harms of the intervention** |  |  |
| How are the benefits and harms of the intervention distributed across the population? Who carries the burden (e.g. all), who benefits (e.g. a very small sub-group)? | In GRADE framework, this may be addressed under 1. Effects, and then further considered when evaluating overall balance of undesirable and desirable effects | The recently updated Cochrane reviews do not provide evidence to enable assessment of whether the balance of benefits and harms in relation to the three topics varies in different population subgroups [8, 32, 33]. |
| **Affordability of intervention** |  |  |
| How affordable is the intervention for individuals, households or communities?  How will it impact household health expenditures, including risk of catastrophic health expenditures and health-related financial risks? | Affordability is considered under 5. Acceptability. | We think this question sits better under consideration of equity. We did not find any direct evidence in relation to affordability of induction of labour for individuals, households or communities. |
| **Accessibility of intervention** |  |  |
| How accessible - in terms of physical as well as informational access - is the intervention across different population groups? | Scope in the framework to consider this question under 4. Equity | The question of physical access has been considered under the sub-criteria “Impact on health equality and/or health equity”. The question of informational access has been considered under Acceptability: Accordance with university human rights standards and principles |
| ***4. Societal implications*** |  |  |
| ***Social impact*** |  |  |
| *What is the social impact of the intervention: Are there features of the intervention that increase or reduce stigma and that lead to social consequences (see Balance of health benefits and harms)?*  *Does the intervention enhance or limit social goals, such as education, social cohesion and the attainment of various human rights beyond health?*  *Does it change social norms at individual or population level?*  *Does it impact research and innovation?* |  | Induction of labour is a simple intervention undertaken in a healthcare setting, where there are few, if any, concerns for sectors beyond health. We decided that the criterium of societal implications (social and environmental impacts) was not relevant for our evidence assessment. |
| ***Environmental impact*** |  |  |
| *What is the environmental impact of the intervention?*  *Does it contribute to or limit the achievement of goals to protect the environment and efforts to mitigate or adapt to climate change?* |  |  |
| **4. Financial and economic considerations** | **3. Resources** |  |
| **Financial impact** |  |  |
| What is the cost of the intervention? | How large are the resource requirements (costs)?  What is the certainty (quality) of the evidence of  resource requirements (costs)? | We have identified limited evidence to answer these questions, and also prepared tables of resource requirements, as per standard WHO EtD documents. |
| What is the overall budget impact of implementing the intervention? |  |  |
| Do cost and budget impacts vary in the short- versus longer-term, and are they sustainable? |  |  |
| **Ratio of costs and benefits** |  |  |
| What is the value-for-money of the intervention, based on an appropriate choice of method, e.g. cost-effectiveness, cost-benefit or cost-utility? | Does the cost effectiveness of the intervention  favour the intervention or the comparison? |  |
| **5. Feasibility** | **6. Feasibility** |  |
| **Need for, usage of and impact on health workforce and human resources** |  |  |
| How does the intervention interact with the need for and usage of the existing health workforce? | Is the intervention feasible to implement? | Workforce/human resources questions presented second in findings for this criteria. |
| Is it likely to impact on these in positive or negative ways, for example by affecting the number or distribution of staff, their skills, responsiveness or productivity? |  |  |
| **Need for, usage of and impact on infrastructure** |  |  |
| How does the intervention interact with the need for and usage of the existing health system infrastructure (e.g. types of health facilities, health information system, medical products and technologies) at national and subnational levels? |  | Infrastructure questions presented first in findings for this criteria. |
| Is it likely to impact on these and their performance in positive or negative ways? |  |  |

# Characteristics of included qualitative studies

| First author | Year | Title | Characteristics | Quality | Source | Rationale for inclusion |
| --- | --- | --- | --- | --- | --- | --- |
| Coates, R | 2019 | Women’s experiences of induction of labour: qualitative systematic review and thematic synthesis | QES of 10 primary studies published between 2010 and 2018 from high-income settings with data gathered through in-depth interviews with a total of 157 women Inclusion: high- and low-risk; IOL for any indication (except fetal death); inpatient & outpatient | High  (reviewer assessment as per protocol) | database search | Per protocol (selected QES) |
| Ezeanochie | 2013 | Women's concerns and satisfaction with induced labour at term in a Nigerian population | Primary qualitative study (Nigeria) of n=252 women; pre/post survey for IOL at term | 100% (QES author assessment using MMAT; Coates D 2020) | Coates, D 2020 | Per protocol (only LMIC primary study in eligible QES/scoping reviews, but not included in selected QES) |
| Henderson | 2013 | Women's experience of induction of labor: a mixed methods study | Primary qualitative study (UK); findings extracted from qualitative thematic analysis of open-ended questions; n=161 responses related to IOL; 37 to 42+ weeks; multiple indications | Moderate (QES author assessment using SRQR; Lou 2018) | Lou 2018 | Per protocol (primary study from eligible QES, but not included in selected QES, with additional findings relating to pain) |
| Nippita | 2017 | Variation in clinical decision-making for induction of labour: a qualitative study | Primary qualitative study (Australia); interviews with 21 midwives & 24 obstetric medical staff across 10 maternity hospitals | 75% (QES author assessment using MMAT; Coates D 2020) | Coates, D 2020 | Per protocol (primary study from eligible scoping review, but not included in selected QES, with only findings relating to implementer acceptability) |
| Westfall | 2004 | The rhetoric of "natural" in natural childbirth: childbearing women's perspectives on prolonged pregnancy and induction of labour | Primary qualitative study (Canada); interviews with 27 women 1-4 mths postpartum; IOL at 42-43 weeks; indication not specified | High (QES author assessment using SRQR; Lou 2018) | Lou 2018 | Per protocol (primary study from eligible scoping review, but not included in selected QES, with additional findings relating to autonomy) |
| Wessberg | 2017 | Being in limbo: Women’s lived experiences of pregnancy at 41 weeks of gestation and beyond – A phenomenological study | Primary qualitative study (Sweden); interviews with 10 women at GA ≥41 weeks; normal pregnancy | High (reviewer assessment using JBI checklist) | Middleton 2020 | Per protocol (primary study from reference list search of 3 Cochrane reviews; relevant population) |

# Participant characteristics in included qualitative studies

| First author | Year | Title | References to participant characteristics |
| --- | --- | --- | --- |
| Brown [in QES Coates R 2019] | 2015 | Women’s experiences of cervical ripening as inpatients on an antenatal ward | "Interviews were offered in the English language only, therefore women who did not read or speak English were also excluded." (p. 220). Participant characteristics not reported with exception of age and parity. |
| Gammie [in QES Coates R 2019] | 2015 | Time’s up! Women’s experience of induction of labour | Inclusion/exclusion criteria not reported. Participant characteristics not reported with exception of parity. |
| Gatward [in QES Coates R 2019] | 2015 | Women’s experiences of being induced for post-date pregnancy | "Anita (I), whose ﬁrst language was Spanish..." (p. 7). Participant characteristics otherwise not reported. |
| Ezeanochie | 2013 | Women's concerns and satisfaction with induced labour at term in a Nigerian population | “About two-thirds (64.3%) of the study population had post-secondary education…” (p. 2) |
| Henderson | 2013 | Women's experience of induction of labor: a mixed methods study | "These 161 women reﬂected the characteristics of respondents generally in terms of age but were disproportionately nulliparous, from afﬂuent areas and more likely to be born in the UK." (p. 1161) |
| Jay [in QES Coates R 2019] | 2018b | In labor or in limbo? The experiences of women undergoing induction of labor in hospital: Findings of a qualitative study | "As a result of cost constraints, it was not possible to employ translators for non- English speakers, thereby excluding this group." "The final sample comprised 21 women, who identified their ethnicity as white British (n = 16), non-white British (n = 1), and white non-British (n = 4). All were married or cohabitating and most were educated to tertiary level." (p. 65) |
| Moore [in QES Coates R 2019] | 2014 | Moving toward patient-centered care: Women’s decisions, perceptions, and experiences of the induction of labor process | Sample reflective of US IOL population; 70% white; 14% Black; 7% Asian; 3% American Indian, Indian, Arab/Middle Eastern; (extracted from Table 1); needed to be English speaking. |
| Murtagh [in QES Coates R 2019] | 2014 | Women’s experiences of induction  of labour for post-date pregnancy | "No restrictions were placed upon participants’ nationality or ethnic origin. However, due to financial constraints the use of an interpreter was not possible and therefore only those who could speak English fluently could be included." (p. 106)  Participant characteristics not reported (apart from parity). |
| Nippita | 2017 | Variation in clinical decision-making for induction of labour: a qualitative study | Participant characteristics not reported (other than profession and length of service in their current hospital). |
| O’Brien [in QES Coates R 2019] | 2013 | A participatory action research study exploring women's understandings of  the concept of informed choice during pregnancy and childbirth in Ireland | "...women whose spoken English prevented them from achieving written informed consent were excluded." (p. 2)  **Country of origin:** Ireland 11 (73.3%), Canada 1 (6.6%), Poland 2 (13.3%), Africa 1 (6.6%) **Education:** Third level 11 (73.3%), Second Level 4 (26.6%) |
| Oster [in QES Coates R 2019] | 2011 | Inpatient versus outpatient cervical priming for induction of labour: Therapeutic landscapes and women’s preferences | "the ability to read and write in English" (p. 380); "Participants were chosen based on a selection matrix of: age, parity, language other than English spoken at home, education, and type of delivery."; "the ﬁnal sample of 16 women was generally representative of the overall cohort with 2/3 of women in the study being primiparous (i.e. pregnant for the ﬁrst time) and highly educated."; "Four (4/16) women spoke a language other than English at home." (p. 381) |
| Reid [in QES Coates R 2019] | 2011 | The home as an appropriate setting for women undertaking cervical ripening  before the induction of labour | "The sample was analysed for level of social deprivation by residence postcode using the Scottish Index of Multiple Deprivation (SIMD), the measure of relative deprivation now used in Scotland (Scottish Executive, 2006). The interviewees did not differ according to SIMD rank, with six of 10 women in both trial groups living in data zones which fell into the lowest two ranks of deprivation. Of the non-responders, 11 of 15 came from the two most deprived ranks." (p. 32) |
| Westfall | 2004 | The rhetoric of "natural" in natural childbirth: childbearing women's perspectives on prolonged pregnancy and induction of labour | "Most (22) were Canadian born; others were born in the United States, Germany or Asia. Most (20) were Caucasian, though a few were Metis (of Aboriginal and French Canadian mixed heritage), Asian, or had some other mixed genetic background. All the participants had completed high school, and the majority (22) had some post-secondary education; ten women held undergraduate and/or graduate degrees. Overall, the sample was slightly more educated than would have been found in a random sample." (p. 1400) |
| Wessberg | 2017 | Being in limbo: Women’s lived experiences of pregnancy at 41 weeks of gestation and beyond – A phenomenological study | "Variations were reached by interviewing women with different background, age, and parity." "Three women had primary and secondary school degrees and seven women had university degree...All participating women spoke Swedish." (p. 3) "The study did not include women from low income groups or from cultures other than the Swedish one, which can be a limitation." (p. 10) |

# References

1. Coates R, Cupples G, Scamell A, McCourt C. Women's experiences of induction of labour: Qualitative systematic review and thematic synthesis. Midwifery 2019;69:17-28

2. Wessberg A, Lundgren I, Elden H. Being in limbo: Women’s lived experiences of pregnancy at 41 weeks of gestation and beyond–A phenomenological study. BMC pregnancy and childbirth 2017;17(1):1-12

3. Ezeanochie M, Olagbuji B, Ande A. Women's concerns and satisfaction with induced labour at term in a Nigerian population. The Nigerian Postgraduate Medical Journal 2013;20(1):1-4

4. Goeree R, Hannah M, Hewson S. Cost-effectiveness of induction of labour versus serial antenatal monitoring in the Canadian Multicentre Postterm Pregnancy Trial. CMAJ : Canadian Medical Association Journal 1995;152(9):1445-50

5. Hannah ME, Hannah WJ, Hellmann J, Hewson S, Milner R, Willan A, et al. Induction of labor as compared with serial antenatal monitoring in post-term pregnancy: a randomized controlled trial. N Engl J Med 1992;326(24):1587-92

6. Grobman WA, Sandoval G, Reddy UM, Tita ATN, Silver RM, et al. Health resource utilization of labor induction versus expectant management. Am J Obstet Gynecol 2020;222(4):369.e1-.e11. doi:10.1016/j.ajog.2020.01.002

7. Grobman WA, Rice MM, Reddy UM, Tita AT, Silver RM, Mallett G, et al. Labor induction versus expectant management in low-risk nulliparous women. N Engl J Med 2018;379(6):513-23

8. Middleton P, Shepherd E, Morris J, Crowther CA, Gomersall JC. Induction of labour at or beyond 37 weeks' gestation. Cochrane Database Syst Rev 2020, Issue 7: Art. No.: CD004945. DOI: 10.1002/14651858.CD004945.pub5. doi:10.1002/14651858.CD004945.pub5

9. Eddy K, Vogel J. Cost-effectiveness evidence for maternal and perinatal health interventions: living scoping review. World Health Organization 2020. osf.io/jwtge. Accessed 5 Jul 2021

10. Kaufman KE, Bailit JL, Grobman W. Elective induction: An analysis of economic and health consequences. Am J Obstet Gynecol 2002;187(4):858-63. doi:10.1067/mob.2002.127147

11. Kaimal AJMDMAS, Little SEMD, Odibo AOMDM, Stamilio DMMDM, Grobman WAMDMBA, Long EFP, et al. Cost-effectiveness of elective induction of labor at 41 weeks in nulliparous women. Am J Obstet Gynecol 2011;204(2):137.e1-.e9. doi:10.1016/j.ajog.2010.08.012

12. Hersh AR, Skeith AE, Sargent JA, Caughey AB. Induction of labor at 39 weeks of gestation versus expectant management for low-risk nulliparous women: a cost-effectiveness analysis. Am J Obstet Gynecol 2019;220(6):590.e1-.e10. doi:10.1016/j.ajog.2019.02.017

13. Adelson PL, Wedlock GR, Wilkinson CS, Howard K, Bryce RL, Turnbull DA. A cost analysis of inpatient compared with outpatient prostaglandin E2 cervical priming for induction of labour: Results from the OPRA trial. Aust Health Rev 2013;37(4):467-73. doi:10.1071/AH13081

14. Wilkinson C, Adelson P, Turnbull D. A comparison of inpatient with outpatient balloon catheter cervical ripening: a pilot randomized controlled trial. BMC Pregnancy Childbirth 2015;15(1):1-9

15. Alfirevic Z, Gyte GML, Nogueira Pileggi V, Plachcinski R, Osoti AO, Finucane EM. Home versus inpatient induction of labour for improving birth outcomes. Cochrane Database of Systematic Reviews 2020, Issue 8: Art. No.: CD007372. DOI: 10.1002/14651858.CD007372.pub4

16. Son SL, Benson AE, Hart Hayes E, Subramaniam A, Clark EAS, Einerson BD. Outpatient Cervical Ripening: A Cost-Minimization and Threshold Analysis. Am J Perinatol 2020;37(3):245-51. doi:10.1055/s-0039-1694791

17. ten Eikelder M, van Baaren GJ, Oude Rengerink K, Jozwiak M, de Leeuw JW, Kleiverda G, et al. Comparing induction of labour with oral misoprostol or Foley catheter at term: cost-effectiveness analysis of a randomised controlled multi-centre non-inferiority trial. BJOG 2018;125(3):375-83. doi:10.1111/1471-0528.14706

18. ten Eikelder ML, Rengerink KO, Jozwiak M, De Leeuw JW, De Graaf IM, Van Pampus MG, et al. Induction of labour at term with oral misoprostol versus a Foley catheter (PROBAAT-II): a multicentre randomised controlled non-inferiority trial. The Lancet 2016;387(10028):1619-28

19. van Baaren GJ, Jozwiak M, Opmeer BC, Oude Rengerink K, Benthem M, Dijksterhuis MGK, et al. Cost-effectiveness of induction of labour at term with a Foley catheter compared to vaginal prostaglandin E2 gel (PROBAAT trial). BJOG 2013;120(8):987-95. doi:10.1111/1471-0528.12221

20. Jozwiak M, Rengerink KO, Benthem M, Van Beek E, Dijksterhuis MG, De Graaf IM, et al. Foley catheter versus vaginal prostaglandin E2 gel for induction of labour at term (PROBAAT trial): an open-label, randomised controlled trial. The Lancet 2011;378(9809):2095-103

21. de Vaan MDT, ten Eikelder MLG, Jozwiak M, Palmer KR, et al. Mechanical methods for induction of labour. Cochrane Database of Systematic Reviews 2019, Issue 10: Art. No.: CD001233. DOI: 10.1002/14651858.CD001233.pub3. doi:10.1002/14651858.CD001233.pub3

22. Alfirevic Z, Keeney E, Dowswell T, Welton NJ, Medley N, Dias S, et al. Methods to induce labour: a systematic review, network meta-analysis and cost-effectiveness analysis. BJOG : an international journal of obstetrics and gynaecology 2016;123(9):1462-70. doi:10.1111/1471-0528.13981

23. State of inequality: reproductive maternal newborn and child health. Geneva: World Health Organization. 2015.

24. WHO recommendations: Induction of labour at or beyond term. Geneva: World Health Organization. <https://apps.who.int/iris/bitstream/handle/10665/277233/9789241550413-eng.pdf?ua=1> (2018)

25. Vogel JP, Souza JP, Gülmezoglu AM. Patterns and outcomes of induction of labour in Africa and Asia: a secondary analysis of the WHO Global Survey on Maternal and Neonatal Health. PLoS One 2013;8(6):e65612

26. Fox H, Topp SM, Lindsay D, Callander E. Ethnic, socio-economic and geographic inequities in maternal health service coverage in Australia. The International Journal of Health Planning and Management. 2021; doi:10.1002/hpm.3277

27. Protheroe J, Brooks H, Chew-Graham C, Gardner C, Rogers A. ‘Permission to participate?’ A qualitative study of participation in patients from differing socio-economic backgrounds. J Health Psychol 2013;18(8):1046-55. doi:10.1177/1359105312459876

28. Primary health care on the road to universal health coverage: 2019 global monitoring report. Geneva, CH: World Health Organization. 2021; (9240004270).

29. Vogel JP, Gülmezoglu AMM, Hofmeyr GJ, Temmerman M. Global perspectives on elective induction of labor. Clin Obstet Gynecol 2014;57(2):331-42

30. Nippita TA, Porter M, Seeho SK, Morris JM, Roberts CL. Variation in clinical decision-making for induction of labour: a qualitative study. BMC Pregnancy Childbirth 2017;17(1):1-9

31. WHO recommendations on antenatal care for a positive pregnancy experience. Geneva, CH: World Health Organization. 2016; (9241549912).

32. Alfirevic Z, Gyte GM, Nogueira Pileggi V, Plachcinski R, Osoti AO, Finucane EM. Home versus inpatient induction of labour for improving birth outcomes. The Cochrane database of systematic reviews 2020;8:CD007372. doi:10.1002/14651858.CD007372.pub4

33. de Vaan MD, Ten Eikelder ML, Jozwiak M, Palmer KR, Davies-Tuck M, Bloemenkamp KW, et al. Mechanical methods for induction of labour. The Cochrane database of systematic reviews 2019;10:CD001233. doi:10.1002/14651858.CD001233.pub3

34. High-Performance Health Financing for Universal Health Coverage. New York, USA: The World Bank. 2019.

35. Westfall RE, Benoit C. The rhetoric of "natural" in natural childbirth: childbearing women's perspectives on prolonged pregnancy and induction of labour. Soc Sci Med 2004;59(7):1397-408. doi:10.1016/j.socscimed.2004.01.017

36. Technical guidance on the application of a human-rights based approach to the implementation of policies and programmes to reduce preventable maternal morbidity and mortality. United Nations Human Rights Council New York. 2012. <https://www.ohchr.org/Documents/HRBodies/HRCouncil/RegularSession/Session21/A-HRC-21-22_en.pdf>. Accessed 23 Aug 2021

37. Tunçalp Ӧ, Were W, MacLennan C, Oladapo O, Gülmezoglu A, Bahl R, et al. Quality of care for pregnant women and newborns—the WHO vision. BJOG 2015;122(8):1045-9

38. Downe S, Finlayson K, Oladapo OT, Bonet M, Gülmezoglu AM. What matters to women during childbirth: A systematic qualitative review. PLoS One 2018;13(4):e0194906-e. doi:10.1371/journal.pone.0194906

39. Seijmonsbergen-Schermers AE, van den Akker T, Rydahl E, Beeckman K, Bogaerts A, Binfa L, et al. Variations in use of childbirth interventions in 13 high-income countries: A multinational cross-sectional study. PLoS Med 2020;17(5):e1003103

40. Guerra GV, Cecatti JG, Souza JP, Faundes A, Morais SS, Gulmezoglu AM, et al. Factors and outcomes associated with the induction of labour in Latin America. BJOG 2009;116(13):1762-72. doi:10.1111/j.1471-0528.2009.02348.x

41. Guerra GV, Cecatti JG, Souza JP, Faúndes A, Gülmezoglu AM, Passini Jr R, et al. Elective induction versus spontaneous labour in Latin America. Bull World Health Organ 2011;89(9):657-65. doi:10.2471/BLT.08.061226

42. World Health Organization Model List of Essential Medicines: 21st List 2019. Geneva, CH: World Health Organization. 2019; (WHO/MVP/EMP/IAU/2019.06).

43. Puchalski Ritchie LM, Khan S, Moore JE, Timmings C, van Lettow M, Vogel JP, et al. Low- and middle-income countries face many common barriers to implementation of maternal health evidence products. J Clin Epidemiol 2016;76:229-37. doi:10.1016/j.jclinepi.2016.02.017

44. Lewin S, Booth A, Glenton C, Munthe-Kaas H, Rashidian A, Wainwright M, et al. Applying GRADE-CERQual to qualitative evidence synthesis findings: introduction to the series. Implementation Science 2018;13(1):1-10

45. Akuamoah-Boateng J, Spencer R. Woman-centered care: Women's experiences and perceptions of induction of labor for uncomplicated post-term pregnancy: A systematic review of qualitative evidence. Midwifery 2018;67:46-56

46. Lou S, Hvidman L, Uldbjerg N, Neumann L, Jensen TF, Haben JG, et al. Women's experiences of postterm induction of labor: A systematic review of qualitative studies. Birth (Berkeley, Calif) 2019;46(3):400-10. doi:10.1111/birt.12412

47. Coates D, Goodfellow A, Sinclair L. Induction of labour: Experiences of care and decision-making of women and clinicians. Women and birth : journal of the Australian College of Midwives 2020;33(1):e1-e14. doi:10.1016/j.wombi.2019.06.002

48. Henderson J, Redshaw M. Women's experience of induction of labor: a mixed methods study. Acta Obstet Gynecol Scand 2013;92(10):1159-67. doi:10.1111/aogs.12211

49. World Health Organization. WHO handbook for guideline development. 2nd ed: World Health Organization; 2014. <https://apps.who.int/iris/handle/10665/145714>.

50. Alonso-Coello P, Oxman AD, Moberg J, Brignardello-Petersen R, Akl EA, Davoli M, et al. GRADE Evidence to Decision (EtD) frameworks: a systematic and transparent approach to making well informed healthcare choices. 2: Clinical practice guidelines. BMJ 2016;353:i2089. doi:10.1136/bmj.i2089
